# Coupling genetic structure analysis and ecological-niche modeling in Kersting’s groundnut in West Africa

**DOI:** 10.1038/s41598-022-09153-5

**Published:** 2022-04-04

**Authors:** Mariam Coulibaly, Rodrigue Idohou, Félicien Akohoue, Andrew Townsend Peterson, Mahamadou Sawadogo, Enoch Gbenato Achigan-Dako

**Affiliations:** 1 Laboratory of Genetics, Biotechnology and Seed Science (GBioS), Faculty of Agronomic Sciences, University of Abomey-Calavi, 01 BP 526 Abomey-Calavi, Republic of Benin; 2 Laboratory of Biosciences, Faculty of Earth and Life Science, University of Ouaga I Pr. Joseph Ki-Zerbo, 03 BP 7021 Ouagadougou, Burkina Faso; 3 Laboratory of Biomathematics and Forest Estimates, Faculty of Agricultural Sciences, University of Abomey-Calavi, BP 1493 Abomey-Calavi, Republic of Benin; 4 Biodiversity Institute, University of Kansas, Lawrence, KS 66045 USA

**Keywords:** Ecology, Evolution, Genetics, Molecular biology, Plant sciences, Climate sciences, Ecology

## Abstract

Orphan legume crops play an important role in smallholder farmers’ food systems. Though less documented, they have the potential to contribute to adequate nutrition in vulnerable communities. Unfortunately, data are scarce about the potential of those crops to withstand current and future climate variations. Using *Macrotyloma geocarpum* as an example, we used ecological niche modeling to explore the role of ecology on the current and future distributions of genetic populations of Kersting’s groundnut. Our findings showed that: (1) the models had good predictive power, indicating that *M. geocarpum’s* distribution was correlated with both climatic and soil layers; (2) identity and similarity tests revealed that the two genetic groups have identical and similar environmental niches; (3) by integrating the genetic information in niche modeling, niches projections show divergence in the response of the species and genetic populations to ongoing climate change. This study highlights the importance of incorporating genetic data into Ecological Niche Modeling (ENM) approaches to obtain a finer information of species’ future distribution, and explores the implications for agricultural adaptation, with a particular focus on identifying priority actions in orphan crops conservation and breeding.

## Introduction

Defining how and where plant varieties will adequately respond to environmental variations is a central topic in plant science research. This is more preoccupying for orphan crops that are largely grown in marginal areas and neglected in the mainstream research agenda^1^. Availability of the genetic resources of those crops is still a challenge for many genebanks at national, regional, and international levels. This situation jeopardizes the sustainable utilization of the plant genetic diversity that can be useful for current and future food systems and secured nutrition^2–4^. Such genetic resources are important for successful cultivars development and selection of economic and agronomic traits and could confer resilience to evolving climate.

In Kersting’s groundnut [*Macrotyloma geocarpum* (Harms) Maréchal and Baudet], a multipurpose staple orphan crop with high nutritional and economic values for smallholder farmers in West Africa^5–9^, the need to solve the ecological suitability of the extant genetic resources arose despite the significant achievements made recently on the germplasm collection, conservation, and characterization. Kersting’s groundnut plays an important role in farming sustainability through its ability to fix atmospheric nitrogen in the soil and enhance soil fertility^10^. Furthermore, It serves in traditional medicine for local populations^6,7,11^. However, the production of Kersting’s groundnut is declining rapidly and the genetic resources were rarely collected and safeguarded for the future generation. In addition, environmental stresses are among the main causes for declining Kersting’s groundnut production from its cultivated areas^5,12^. Though Kersting’s groundnut has relatively good adaptation to low-input conditions^13,14^, increased frequency of drought, intense precipitations, elevated temperatures, and increased salt and heavy metals in soils will often be accompanied by increased infestation by pests, and pathogens, are expected to limit the plant growth and productivity, and consequently the crop’s yield and production^15^.

Recent studies revealed a low variation within the species^10,16^ that limits the extent of its genetic diversity and cultivated zones. Kersting’s groundnut counts six landraces set mostly within three agroclimatic zones; Northern-Guinean (NG), Northern-Sudanian (NS), and Southern-Sudanian (SS) of Benin, Burkina Faso, Ghana, and Togo with the predominance of genetic resources and diversity in the Southern-Sudanian zone^5,12^. Overall, the area of cultivation and adaptation of landraces differ among agroclimatic regions. The Black landrace was largely collected in the Northern-Sudanian environmental conditions and was widely preferred, cultivated, and maintained by farmers^12^. The White landrace was widely grown in the Northern-Guinean transition zone of Benin^5,6^ while less cultivated in Burkina Faso^12^ and absent in other countries of West-Africa. The production of the Brown landrace was specifically limited to the Ghana farming system^12^.

Kersting’s groundnut landraces are the direct results of farmer selection, cultivation, and maintenance over the centuries. This continual adaptation of the crop to smallholders farming conditions could continue to play a role in adapting production to climate change. Also, local adaptation of landraces could vary in their climatic response and requirement and therefore, may spread differentially under evolving environmental conditions^17^. To find an adequate preferendum where the species can thrive, it has become crucial to approximate the potential distribution of the crop and its genetic resources.

Unfortunately, with the rapid evolution in climate conditions and the further introduction and adoption of new cash crops with high economic importance, local seed systems alone will likely be insufficient to ensure the endurance of the crop genetic resources and diversity. In these conditions, applying ecological research is required to inform conservation and management decisions to mitigate a species genetic erosion^18^, as Kersting’s groundnut at National and Regional levels. Ecological niche modeling (ENM) can identify the environmental parameters that can impact a species’ distribution and project its potential distribution area onto new environmental surfaces to examine the effect of present or future environmental change^19,20^.

Several statistical and mechanistic techniques proved effective in quantifying niches and spatial distribution of natural and cultivated species^21–25^. The basic modeling framework of species distribution models (SDMs) in general has been criticized on a number of gaps, such as ignoring heterogeneity in population and genetic structure in different parts of a species geographical range^26^. However, many species are organized into differentiated genetic lineages across their geographical ranges^27,28^ and populations differ in their adaptive potential to respond to environmental change^29^. Studies proved that incorporating molecular data into SDMs represents an important step forward for modeling the effects of climate change on species geographical ranges^30–32^.

In the case of Kersting’s groundnut^33^, much uncertainty remains concerning the ability of the crop to withstand the changing climate, suggesting that there is a clear need to comprehensively analyze the response of the crop diversity under new environmental conditions of the coming decades.

The present study was undertaken to determine the environmental conditions in which KG will need to be cultivated in the coming decades and to utilize this information to prioritize genetic resource conservation and breeding efforts. Recent molecular studies involving 281 individuals from Benin and Togo identified two major genetic clusters of KG and these two groups were distributed across Southern-Sudanian and Northern-Guinean agroclimatic zones^16^. Kafoutchoni, et al.^34^ also assessed the genetic structure of the species through the GBS approach with 217 individuals, and Discriminant analysis of principal components (DAPC) and found eight genetically distinct groups from five origins. In this context, the following questions are of high interest: do the agroclimatic niches of KG vary with climate changes? Would KG genetic groups differ in their ability to respond to present and future climatic scenarios?

This study examines the response of orphan crops to future climates by using genetic information and ecological niche modeling approach (gENMs) using KG as an example. Therefore, we combined KG population genomics data with ecological niche modeling: (1) to analyze the relationship between climate factors and species populations distribution in agroclimatic zones of Burkina Faso, Benin, Ghana, and Togo, and (2) to predict areas that would be suitable for the species and genetic populations under the future scenarios. We hypothesized that: (1) the future climates will impact the distribution of KG, and (2) genetically distinct populations of orphan crops would respond differently to climate change.

## Results

### Population structure and admixture

**Figure 1:**
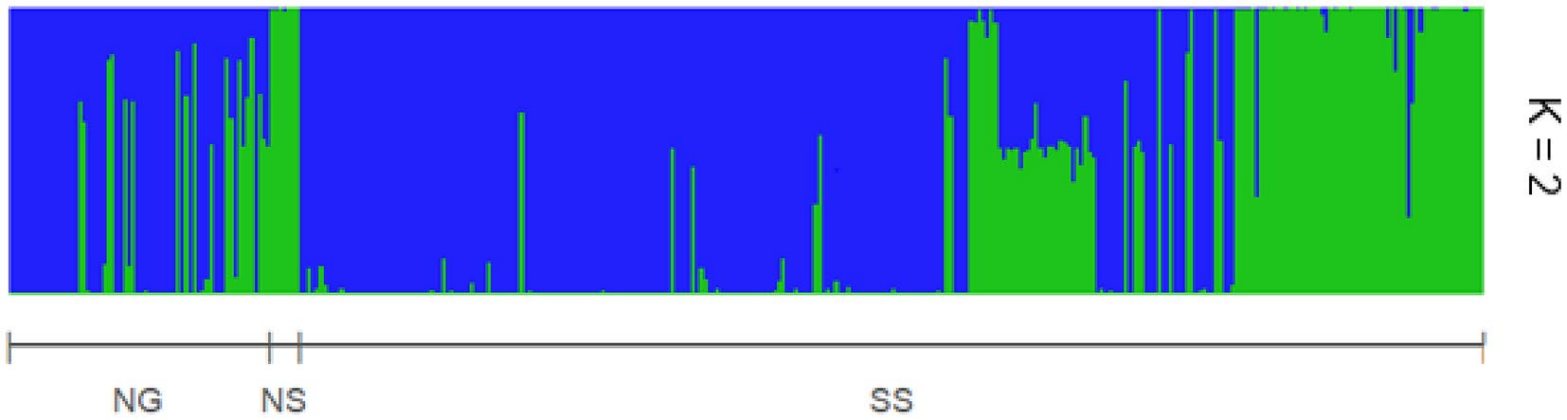
Structure diagram showing the delineation of Kersting’s groundnut individuals into two genetic populations (K = 2), Pop1 (Blue) and Pop2 (Green). Vertical lines represent individuals within populations and those with more than one color share genetic information with other populations. The horizontal line shows the distribution of the populations across agroclimatic zones (Northern-Guinean NG, Northern-Sudanian NS, and Southern-Sudanian SS).

Despite low levels of diversity (He = 0.021 and Ho = 0.0053, p.value ≤ 0.001), Kersting’s groundnut populations remained genetically well differentiated. Admixture models with a putative number of tested genetic clusters (K) from one to five, showed that the most likely number of inferred members was 2 with ΔK = 101.917 (Fig. S1: Evanno output plots; Table S1). The classification of the 361 accessions into populations based on the model-based structure (Fig. 1) showed that more than 65% of individuals belonged to the Pop1 (in blue colour, N = 231).

Table 1 showed the distribution of populations across agroclimatic zones. The majority of Kersting’s groundnut individuals were collected in the Southern-Sudanian zone (< 80%); 83.117% and 75.385% of individuals of Pop1 and Pop2, respectively collected in this zone. All the accessions collected in the Northern-Sudanian zone belonged to the Pop2. In the Northern-Guinean zone, 19.481% of accessions belonged to Pop1 and 14.615% were included in Pop2.

**Table 1:** Number (N) and proportions (Freq) of accessions for each genetic Group per Landrace and agroclimatic zone.

	Features of populations	Pop1		Pop2	
Group by		N	Freq (%)	N	Freq (%)
Landraces seeds coat color	Black	12	5.195	49	37.692
	Brown greyed orange eye	0	0	39	30
	Red	2	0.866	17	13.077
	White	223	96.537	4	3.077
	White black eye	0	0	14	10.769
	White greyed orange eye	0	0	1	0.769
Agroecological zones	Northern-Guinean	2	19.48	19	14.615
	Northern-Sudanian	0	0	7	5.385
	Southern-Sudanian	192	83.117	98	75.385
Totals		237	100	124	100

**Figure 2:**
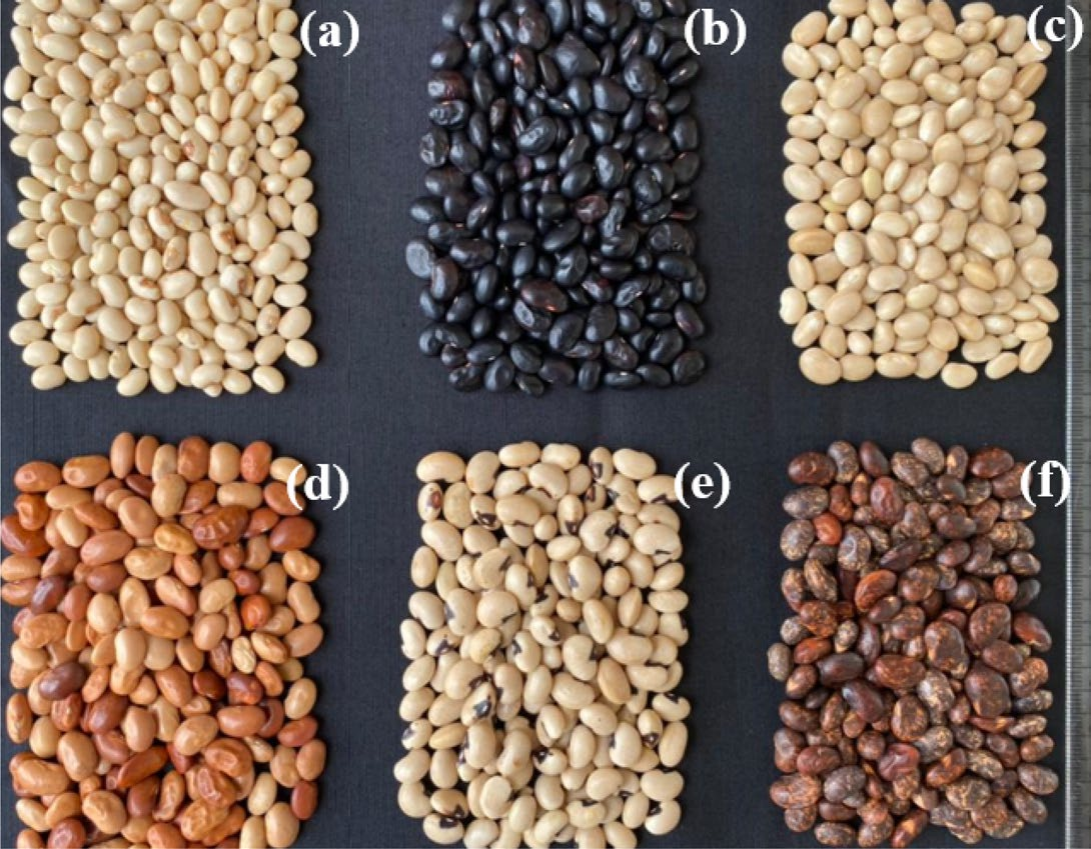
Kersting’s groundnut seed coat colors. (**a**) White mottled with greyed orange eye (**b**), Black, (**c**) White, (**d**) Red, (**e**) White mottled with black eye, (**f**) Brown mottled with greyed orange eye.

The random distribution of landraces (based seed coat colour, Fig. 2) into the genetic populations indicated that Pop1 was mostly characterized by the White landrace (97.403% of individuals in Pop1). On the other hand, the Pop2 was composed of all the six landraces included in this study with a predominance of Black (43.846%) and Brown ones (30%).

The analysis of genetic distance revealed relatively strong genetic differentiation among the two distinct groups of KG with a pairwise Fst value of 0.583 (Table 2). The estimated values of gene diversity (Hs) showed an overall low genetic divergence between individuals within each population (0.017). Meanwhile, the heterozygosity analysis indicated that the genetic divergence between individuals in Pop2 was relatively greater comparing to Pop1.

**Table 2:** Analysis of molecular variance (AMOVA), and genetic variation within and among populations.

Source of variation	Df	SS	MS	Variation (%)	Pairwise Fst	He	Ho	Hs	P-value
Within pop	3	248.276	82.756	4.958		0.021	0.0053	0.017	< 0.001
Between pop	1	2879.945	2879.945	47.895	0.583				< 0.001
Pop1						0.005		0.004	< 0.001
Pop2						0.029		0.029	< 0.001

*DF* degree of freedom, *SS* sum of squares, *MS* expected mean squares, *%* percentage of the variance, *He* expected heterozygosity, *Ho* observed heterozygosity.

*P*-value: significance tests after 1000 permutations.

### Occurrences dataset construction

**Figure 3:**
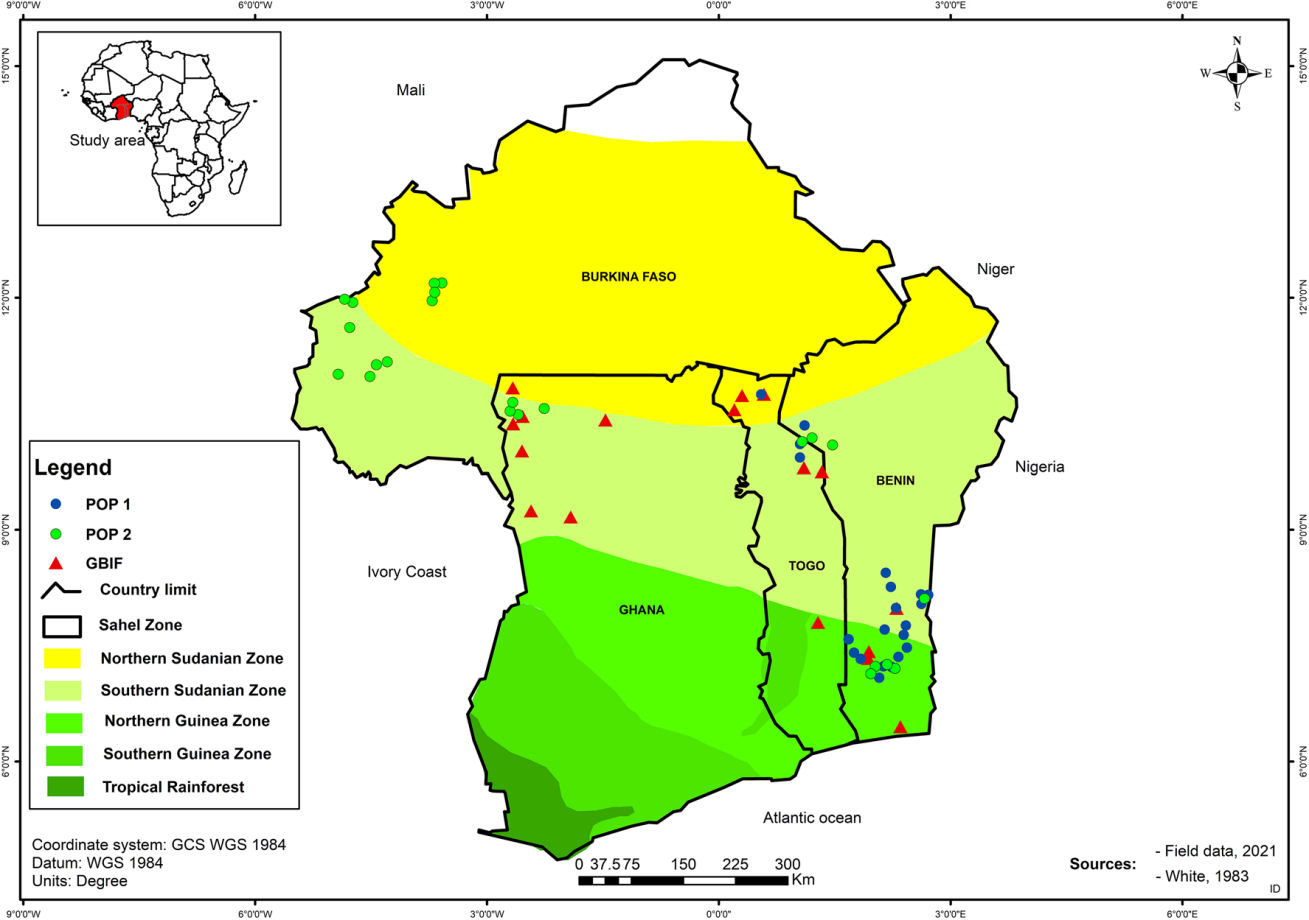
Map showing the distribution of Kersting’s groundnut across agroecological zones of Burkina Faso, Benin, Ghana, and Togo: species collections points (circles) and the locations points downloaded from GBIF (triangles) used in the ecological niche modeling. Genetic groups are depicted by the blue (Pop1) and green (Pop2) colours. Software used: ArcGIS v. 10.7.1.

Figure 3 shows Pop1 distributed across two agroclimatic zones, the Northern-Guinean and Southern-Sudanian zones whereas the Pop2 and Global Biodiversity Information Facility (GBIF) points were represented in all agroclimatic zones. At the country level, Pop2 was distributed across the four countries while Pop1 was located in Benin and Togo.

### Niche overlap and conservatism among *M. geocarpum* and genetic populations

#### Niche similarity between KG and Pop1

**Figure 4:**
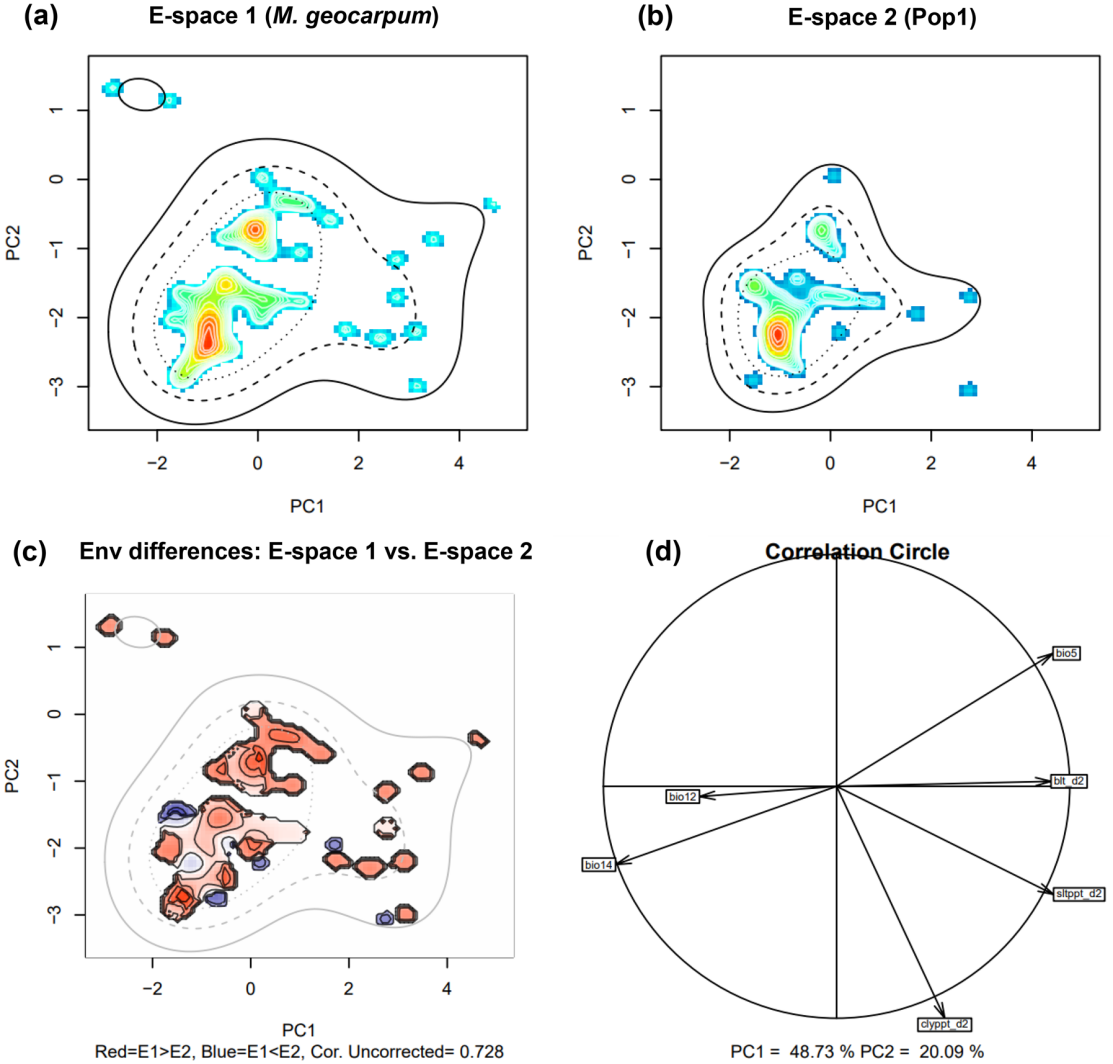
Niches of the two compared groups (*M. geocarpum* and genetic Pop1) in two dimensional E-space. Graphs (**a**) and (**b**) represent the niches of the species and Pop1, respectively along the first two axes of the PCA. The occurrences are represented by kernel density isopleths, red colour indicates high density and blue colour indicates low density. Solid and dotted contour lines illustrate 100% and 50% of the available background (environmental space). (**c**) is the diference in the E-space of the two groups, and Niche E-space Correlation Index (NECI). NECI determines if one should correct the occurrence densities of each group by the prevalence of their environments in their range for equivalency and background tests. For high NECI (> 0.5) groups occupied niches are recommended to be corrected by the frequency of E-space in accessible environments to reduce the chances of committing type 1 errors, and (**d**) is the correlation circle based on the two principal components of the environmental input data. *bio5* max temperature warmest month, *bio12* mean annual rainfall, *bio14* rainfall driest month, *blt_d2* bulk density in kg/cubic-meter for 10 cm depth, *clyppt_d2* soil texture fraction clay at 10 cm depth, *Sltppt_d2* soil texture fraction silt in percent for 10 cm depth.

The quantification of the niches of species (Fig. 4a) and genetic Pop1 (Fig. 4b) in environmental space indicated that more individuals were loaded in PC2 while low number on PC1. Furthermore, the principal Components Analysis (PCA) showed that the first two axes explained 68.82% of the environmental (E-space) variation (PC1 = 48.73% and PC2 = 20.09%) between the species and genetic Pop1. The diference between the E-space of the two groups is illustrated by Fig. 4c. Climate variables correlated with the two principal components were mean annual rainfall (bio12), rainfall driest month (bio14), bulk density in kg/cubic-meter for 10 cm depth (blt_d2) and soil texture fraction clay at 10 cm depth (clyppt_d2) (Fig. 4d: PC1 in horizontal axis and PC2 in vertical axis). The values of the Potential Niche Truncation Index (PNTI) were very low for the species (0.09, Fig. S2a) and Pop1 (0.03, Fig. S2b). These values are below the suggested range of values showing either moderate risk (0.15–0.3) or high risks (0.3) for wich the observed niches do not represent the fundamental niches^35^. In addition, We found a non-signifcant niche equivalency test statistic (D = 0.311, p = 0.990), and significant background test statistic (p = 0.009, Fig. S2c–e).

#### Niche similarity between KG and Pop2

**Figure 5:**
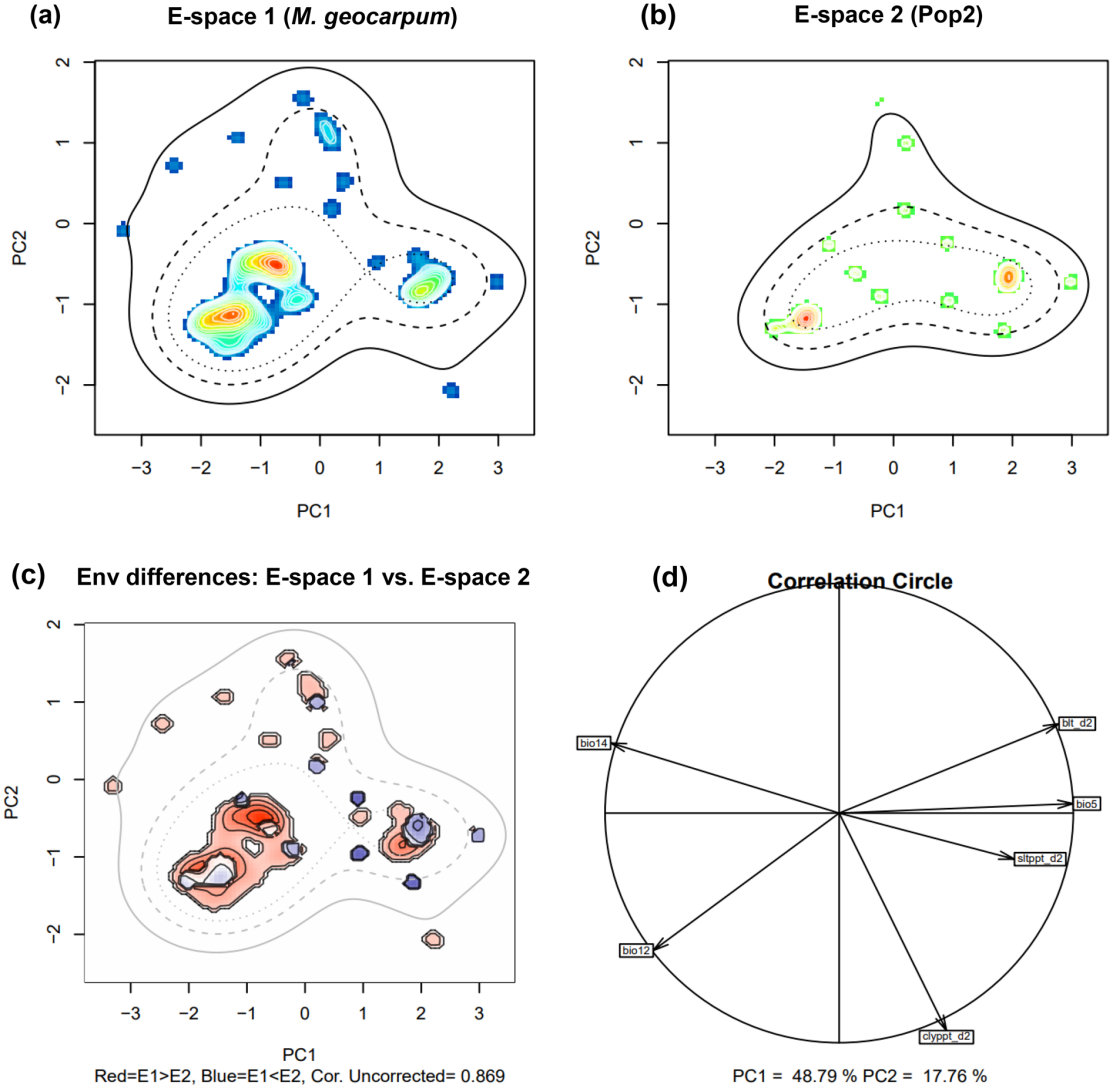
Niches of the two compared groups (*M. geocarpum* and genetic Pop2) in two dimensional E-space. Graphs (**a**) and (**b**) represent the niches of species and Pop2, respectively along the first two axes of the PCA. The occurrences are represented by kernel density isopleths, red colour indicates high density and blue colour indicates low density. Solid and dotted contour lines illustrate 100% and 50% of the available background (environmental space). (**c**): is the diference in the E-space of the two groups, and Niche E-space Correlation Index (NECI). NECI determines if one should correct the occurrence densities of each group by the prevalence of their environments in their range for equivalency and background tests. For high NECI (> 0.5) groups occupied niches are recommended to be corrected by the frequency of E-space in accessible environments to reduce the chances of committing type 1 errors, and (**d**) is the correlation circle based on the two principal components of the environmental input data. *bio5* max temperature warmest month, *bio12* mean annual rainfall, *bio14* rainfall driest month, *blt_d2* bulk density in kg/cubic-meter for 10 cm depth, *clyppt_d2* soil texture fraction clay at 10 cm depth, *Sltppt_d2* soil texture fraction silt in percent for 10 cm depth.

The quantified niches in environmental spaces of the species and Pop2 showed that the individuals of the species and Pop2 were mostly loaded on the PC1 (Fig. 5a,b). The diference between the E-space of the two groups is illustrated by Fig. 5c. The PCA analysis revealed that 66.55% of the variance (PC1 = 48.79% and PC2 = 17.76%) in environmental data input can be represented in a two dimensional E-space (Fig. 5d). Climate variables correlated with the first two axis included the max temperature warmest month (bio5), rainfall driest month (bio14), and bulk density in kg/cubic-meter for 10 cm depth (blt_d2). For the species and genetic Pop2, the PNTI value was low and showed no niche truncation (Fig. S3a,b). Our results showed a non-signifcant niche equivalency test statistic (D = 0.342, p = 0.871) indicating identical environmental niches of *M. geocarpum* and Pop2 (Fig. S3c,d). We obtained a signifcant background test statistic (p = 0.014) (Fig. S3e), which indicates that the species and genetic Pop2 niches were similar.

#### Niche similarity between Pop1 and Pop2

**Figure 6:**
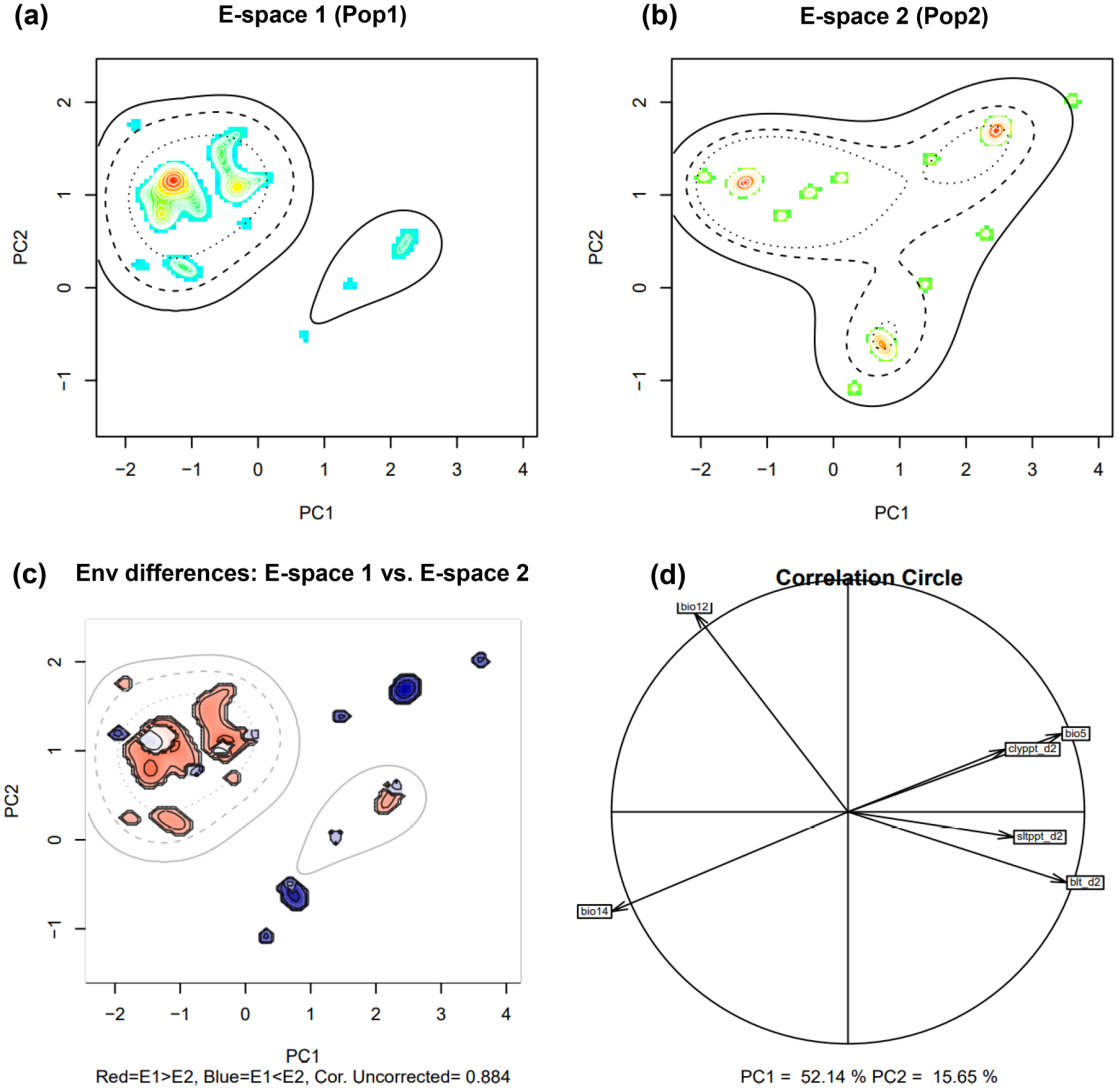
Niches of the two genetic populations in two dimensional E-space. Graphs (**a**) and (**b**) represent the niches of Pop1 and Pop2, respectively along the first two axes of the PCA. The occurrences are represented by kernel density isopleths, red colour indicates high density and blue colour indicates low density. Solid and dotted contour lines illustrate 100% and 50% of the available background (environmental space). (**c**): is the diference in the E-space of the two populations, and Niche E-space Correlation Index (NECI). NECI determines if one should correct the occurrence densities of each population by the prevalence of their environments in their range for equivalency and background tests. For high NECI (> 0.5) genetic populations occupied niches are recommended to be corrected by the frequency of E-space in accessible environments to reduce the chances of committing type 1 errors, and (**d**) is the correlation circle based on the two principal components of the environmental input data. *bio5* max temperature warmest month, *bio12* mean annual rainfall, *bio14* rainfall driest month, *blt_d2* bulk density in kg/cubic-meter for 10 cm depth, *clyppt_d2* soil texture fraction clay at 10 cm depth, *Sltppt_d2* soil texture fraction silt in percent for 10 cm depth.

The quantification of the two genetic populations niches showed that the indiciduals were mainly loaded on PC1 axis (Fig. 6a,b) with Fig. 6c showing the diference between the E-space of the two groups. Moreover, when examining the results of PCA analysis, we found that the first two axes explained 67.79% of the (PC1 = 52.14% and PC2 = 15.65%) in environmental variations between the two genetic populations (Fig. 6d). The PCA correlation circle showed that max temperature warmest month (bio5), soil texture fraction clay at 10 cm depth (clyppt_d2), bulk density in kg/cubic-meter for 10 cm depth (blt_d2), and Soil texture fraction silt in percent for 10 cm depth (Sltppt_d2) were the environmental variables correlated with the two principal components. The PNTI value (< 0.15) indicated that the niches occupied by the two genetic populations represent the fundamental niches of these groups (Fig. S4a,b).We obtained a non-signifcant niche equivalency test statistic (D = 0.213, p = 0.792), and significant background test statistic (p = 0.015, Fig. S4c–e). These results indicates identical and similar environmental niches of the genetic populations.

### Ecological niche modeling

#### Climatic variables analogy

**Figure 7:**
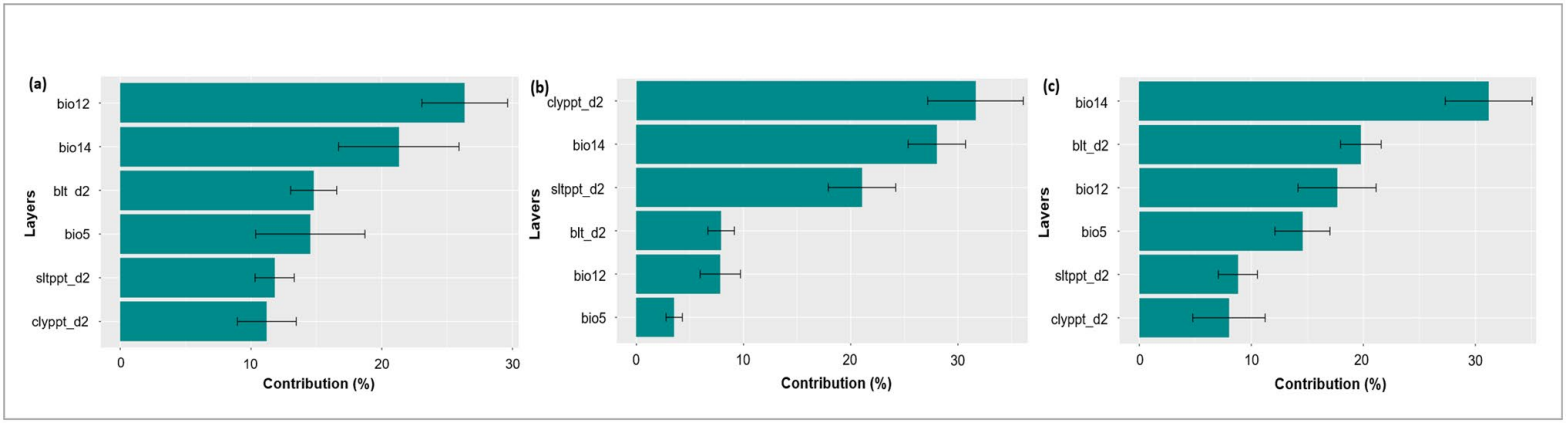
Environmental contribution for the species (**a**) and genetic Pop1 (**b**) and Pop2 (**c**). *bio5* max temperature warmest month, *bio12* mean annual rainfall, *bio14* rainfall driest month, *blt_d2* bulk density in kg/cubic-meter for 10 cm depth, *clyppt_d2* soil texture fraction clay at 10 cm depth, *Sltppt_d2* soil texture fraction silt in percent for 10 cm depth.

Figure 7 shows the importance of the six selected environmental variables in terms of their contribution to each model. When examining the important variables associated with the occurrences data, the three most important variables for prediction models of *M. geocarpum* (Fig. 7a) and Pop2 (Fig. 7c) were the mean annual rainfall (bio12), rainfall driest month (bio14), and bulk density in kg/cubic-meter for 10 cm depth (blt_d2). On the other hand, for Pop1 (Fig. 7b), soil texture fraction clay at 10 cm depth (clyppt_d2), soil texture fraction silt in percent for 10 cm depth (Sltppt_d2), and rainfall driest month (bio14) had the greatest contributions to the model. In contrast, clyppt_d2 and Sltppt_d2 were far less important for model 1 and model 3.

#### Models’ outputs and performance evaluation

The results in Table 3 showed that the environmental predictive models displayed high predictive power for the three models. We found that the ENM for KG location points without genetic information was significant, with pROC scores of 1.294 ± 0.144 (t-test, p < 0.001). The Pop1 and Pop2 models had the highest pROC scores of 1.755 ± 0.115 and 1.525 ± 0.110, respectively. These populations exhibited the best models performance with the lowest AICc scores (Pop1 = 571.670, and Pop2 = 696.185, Table 3). Additionally, these best-supported models had mean omission rates of 0.039 (Pop1) and 0.085 (Pop2) for the 10th percentile training presence. The AUC values were also high for the three niche models: *M. geocarpum* had a mean test AUC of 0.916 ± 0.027, the pop1, 0.979 ± 0.009, and Pop2, 0.926 ± 0.023, indicating that occurrences points were strongly differentiated from background locations, so model distributions were not random.

**Table 3:** Maxent results showing the parameters measuring the three models’ performance and the partial receiver operating characteristic curve (pROC) for each model.

Species/population	Occ	pROC	AUC	Omission rate	AICc	TPLT
*M. geocarpum*	53	1.294 ± 0.144	0.916 ± 0.027	0.093	1362.357	0.313
Pop1	24	1.755 ± 0.115	0.979 ± 0.009	0.039	571.67	0.350
Pop2	26	1.525 ± 0.110	0.926 ± 0.023	0.085	696.185	0.386

*Occ* number of occurrences, *pROC* partial receiver operating characteristic curve, *AUC* area under the receiver operating characteristic curve, *AICc* corrected Akaike Information Criterion, *TPLT* 10 percentile Training Plogistic Threshold.

#### Impact of climate change on Kersting’s groundnut and genetic populations

**Figure 8:**
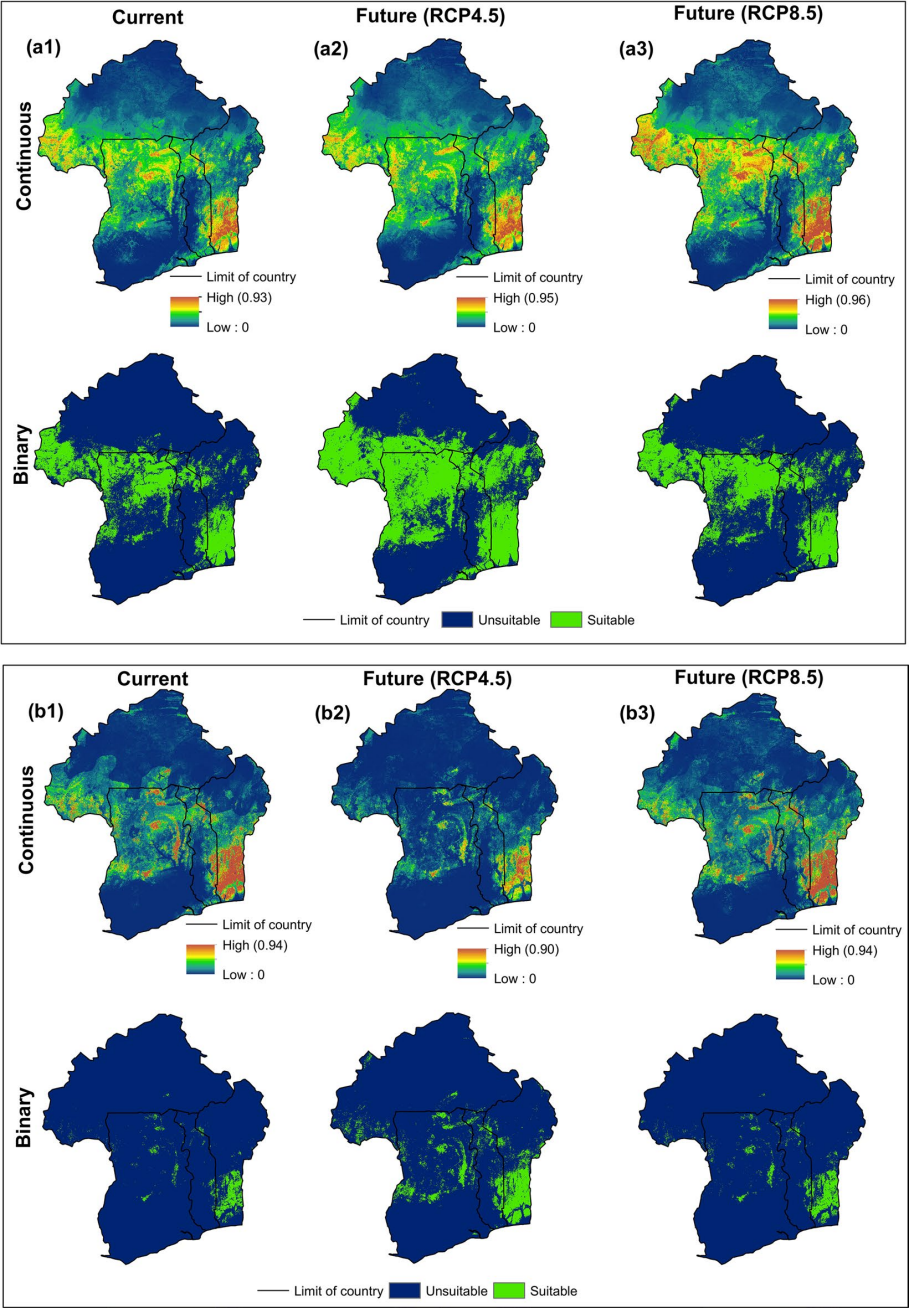

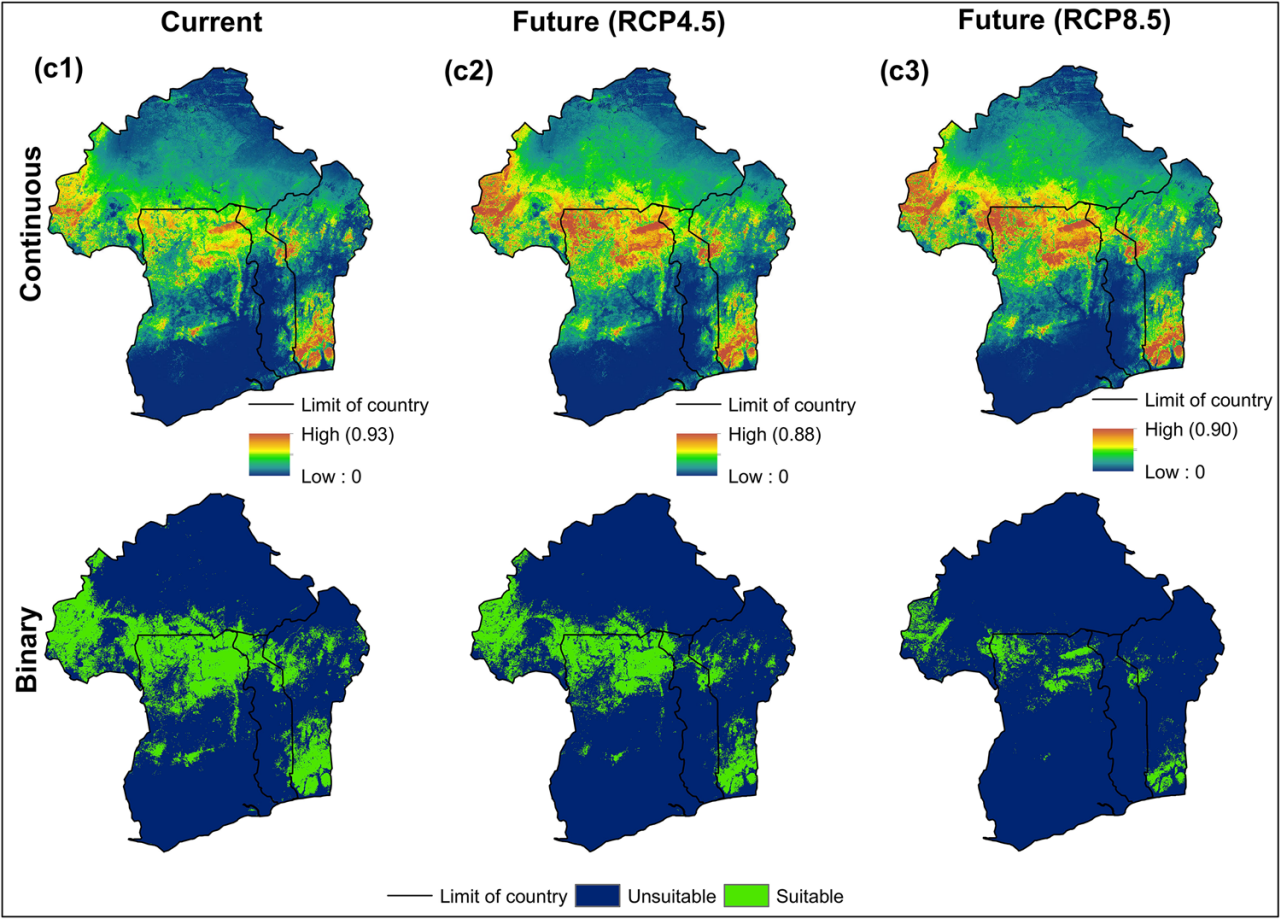
Binary and continuous maps showing the spatial distribution of *M. geocarpum* (**a1**–**a3**) and genetic Pop1 (**b1**–**b3**) and Pop2 (**c1**–**c3**) in present and the future days. Each model was set to thresholds with the 10 percentile training presence values to produce continuous and binary raster maps for current and future scenarios in ArcGIS v. 10.7.1.

Based on the 10 percentile training thresholds (Table 3), the defined calibration areas (Fig. 8) revealed large areas with suitable conditions for the cultivation of KG and genetic populations in the present day (Table S2), except for Pop1. The ENMs predicted broader suitable areas for KG (21.578%) and Pop2 (23.696%), while suitable climatic conditions for Pop1 were restricted to a much smaller area (2.423%).

The Maxent model for the species predicted a large area of suitable conditions across the three agroclimatic zones, Southern Sudanian (SS), Northern Sudanian (NS), and Northern-Guinean (NG) (Fig. 8a1). The SS and NG zones were the areas forecasted to have high suitable climatic conditions for species production. For the Pop1, the areas predicted to have high likely cultivability conditions were concentrated in the NG zone of Benin, but very less and sparsely distributed in the SS zone (Fig. 8b1). The Pop2 was projected across the three studied agroclimatic zones of the four countries (Fig. 8c1).

Analyses based on projecting the final models into the future (2055) scenarios revealed varied patterns in KG and genetic populations cultivable areas (Fig. 8, Figs. S5, S6, and S7). Under the two future climatic scenarios RCP4.5 and RCP8.5, an increase in the species cultivable areas for about 91.137% and 16.567%, respectively were observed due to the decrease of the non-suitable areas (Fig. 8a2,a3, and Fig. S5). The expansion of this area was observed mainly in the NG zone further Southern of Benin, and Togo.. On the other hand, the NG zone of Southern and Central Benin and Togo, and the SS and NS zones of the four countries became climatically unsuitable to crop production.

Important increases were observed in the potential cultivable areas of the Pop1 under future climatic scenarios (> 500% under RCP 4.5 and ≈ 50% under RCP 8.5) (Fig. 8b2,b3, and Fig. S6). This trend was observed across the three agroclimatic zones of the four countries under scenario RCP 4.5. However, under the scenario RCP 8.5, the cultivable areas decreased in the Northern and Southern Guinean zones of Ghana. There was also a slight decrease in the suitable areas of production in some areas of the Northern-Guinean and Southern-Sudanian zones Benin, Burkina Faso, and Togo. The Pop2 showed to be the more vulnerable to future scenarios as the suitable areas significantly decreased (65.758% under RCP4.5 and 76.941% under RCP8.5), while the unsuitable ranges increased (Fig. 8c2,c3, and Fig. S7). Meanwhile, this genetic group is projected to gain in very small suitable areas in the Northern Guinean zone further in the South of Benin, Togo, and Ghana under the severe scenario of RCP 8.5.

**Figure 9:**
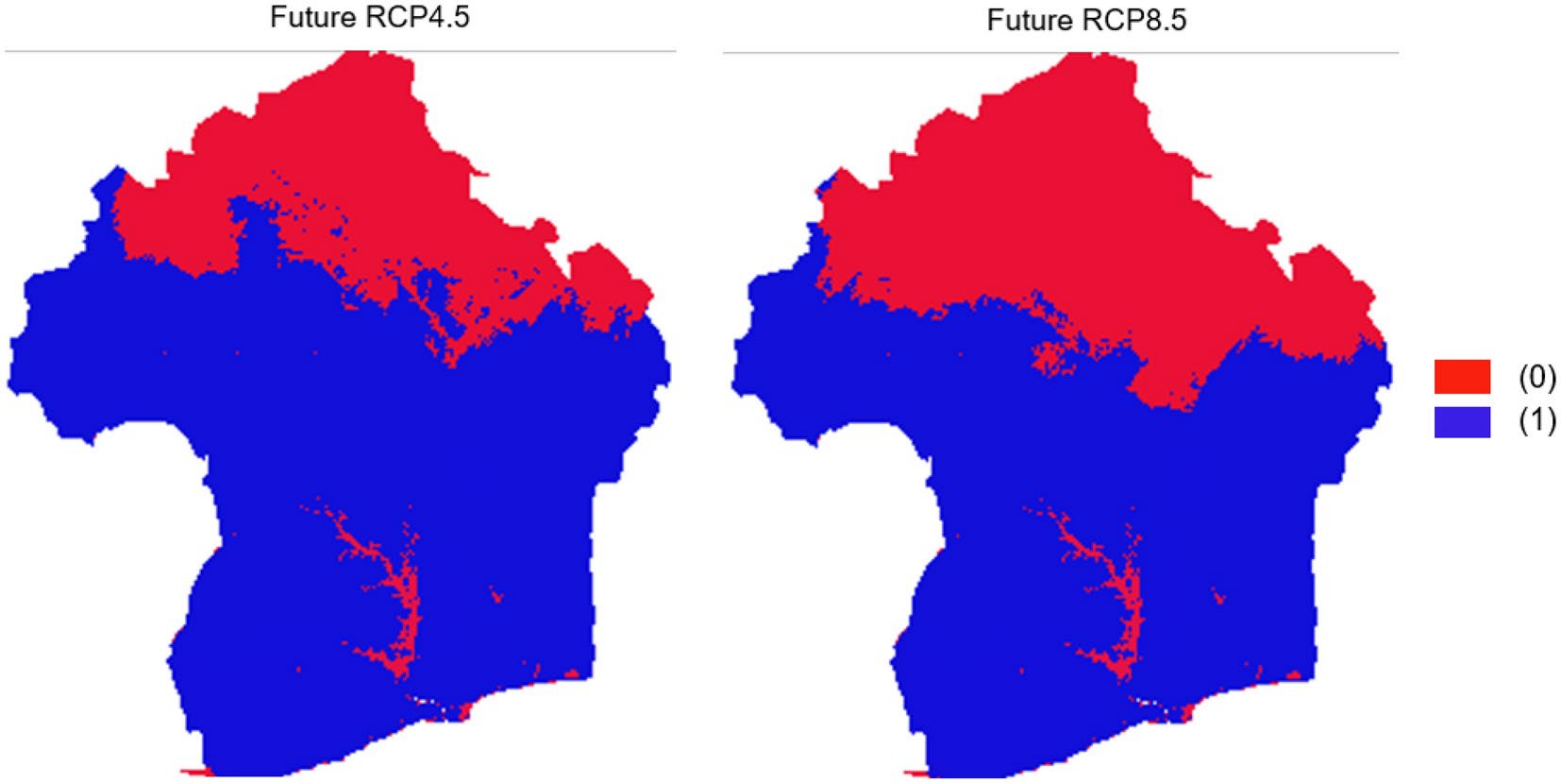
Output of the mobility-oriented parity analysis of KG from ‘ntbox’; 0 (Red) represents strict extrapolation areas meaning complete dissimilarity between calibration environments and extrapolation regions in the future; 1 (Blue) area similar to areas where the models have been calibrated. Software used: ArcGIS v. 10.7.1.

The MOP results indicated that the calibration environments were dissimilar from the extrapolative conditions in the two future scenarios of 2055 (Fig. 9). Figure 9 showed that the Sahel zone in Burkina Faso and the Northern-Sudanian climatic conditions of eastern Burkina Faso, and further North of Benin are unlikely to be suitable for the species cultivation in real life.

## Discussions

By incorporating genetic information in ENM, this study provided evidence that the two genetically distinct groups in Kersting’s groundnut respond differently to climate changes though distributed in similar environmental niches, and suggested implications for sustainable management and breeding perspectives under current and future climate variations.

### Current potential distribution ranges of Kersting’s groundnut and genetic groups

We used two approaches to model KG potential environmental niches across agroclimatic zones of four West African countries: a traditional species ENM using species-wide cultivated range, and intraspecific models based on genetic information. We found that decomposing a species into intraspecific genetic groups increases our understanding of the potential distribution of KG, which is consistent with results from previous studies incorporating genetic information into ENM^30,31,36,37^.

### Environmental variables contribution

Our approach allowed to identify six environmental variables correlated with the potential distribution ranges of the species and populations. The max temperature warmest month (bio5), mean annual rainfall (bio12), and rainfall driest month (bio14) were the climatic variables mostly involved in fitting the three models. The ecological weight that precipitations may have in KG’s distribution is also supported by a recent ethnobotanical study where farmers identified drought and high moisture during the reproductive stage as the main factors hindering species production^5,12^. Tamini also showed that the different sowing dates and seasons with varied temperatures influenced KG growing cycle by delaying or accelerating the flowering time. To cope with these weather issues, farmers managed their cropping calendar by advancing (in June) or delaying (in August) the date of planting KG. The change in temperature and moisture levels are not only determined by climatic parameters but may also lead to a change in the absorption rate of water, fertilizers, and other minerals in soils, which determine yield output^38,39^. Therefore, the consideration of both climate and soil type was essential to better predict KG and genetic groups’ distribution. The physical properties of soil such as bulk density of soil in kg / cubic-meter for 10 cm depth (blt_2), soil texture fraction clay at 10 cm depth (clyppt_d2), and soil texture fraction silt in percent for 10 cm depth (Sltppt_d2) also play a relevant role in KG and populations distribution. Kouelo et al.^40^ reported that the soil texture and preparation (type of tillage) influenced the crop productivity in Benin. Although applying ENM to intraspecific genetic groups allowed the detection of environmental variables, a multidisciplinary approach involving agronomists and biologists would allow a clear understanding of the weight that each of these components may have on crop growth and development.

### Niche equivalency and similarity

We found identical and similar environmental niches for the species and the two genetic populations, suggesting no variation in their environmental niches and adaptation to climate conditions. Our results did not support the view that a given species could be considered as an assemblage of genetic units differing in their spatial distribution^31,36^. Populations of KG are both cultivated in the Northern-Guinean and the Southern-Sudanian zones of Benin, characterized by bimodal (rainfall ≤ 1500 mm) and unimodal (rainfall ≤ 1100 mm) growing seasons, respectively. Although Pop 2 is grown also in the Northern-Sudanian zone, where rainfalls are relatively low (rainfall ≤ 900 mm), very few number of occurences (7) from this region were used in the model. Therefore, they may not show variations in their environmental niches and adaptation to different climate conditions, as previously reported by Wellenreuther, et al.^41^ who analyzed the ecological causes of the ranges limits and the coexistence of two congeneric damselflies (*Calopteryx splendens* and *C. virgo*).

In contrast, Maxent’s outputs revealed that climatic niches of the two genetic populations of *M. geocarpum* are relatively different in the distribution areas. The species and genetic populations occupied the distribution ranges but do not respond similarly to the environmental conditions. Indeed, KG individuals displayed difference in their performance when evaluated under the same environmental conditions^42–44^.

### Predicting evolutionary change based on genetic variation

Our results, found evidence of variability in the species response to future environmental conditions, confirming the assumption that changes in climate will influence the environmental niches of KG species and populatons. This corroborates many other studies which showed the impact of future climatic conditions on crops production, such as cereals, legumes, vegetables^22–24,45,46^.

We also combined genetic information to ENM to test the hypothesis that genetic divergent groups would respond differently to environmental change. The projection under future climatic scenarios RCP4.5 and RCP8.5 indicate that the response of Kersting’s groundnut and genetic groups varied significantly across agroclimatic zones. Our results confirmed the idea that different genetic clusters potentially showed adaptive variation to different abiotic conditions within the geographical range of the species. Globally, although Central Benin and Northern Togo are presumed to be a centre of origin for the crop, we observed a loss in suitable areas for the species production. The same trend was observed in other countries were the crop has been grown for thousand of years. Similarly, Ikeda et al.^31^ tested and demonstrated the hypothesis that species locally adapted to current environments are likely to become maladapted in the future. The same trends were reported on other cereals such as maize, wheat, sorghum, and barley which tend to decrease their area of suitability in their centres of origin^47^. Out of the 361 individuals included in this study, 101 belong to the genetic Pop2, and counts all the landraces grown—diversity based on seed coat color^5,12^. According to the conclusion of Vigouroux, et al.^48^ who described the evolution and adaptation of pearl millet in West Africa, such diversity was supposed to confert adaptation to climate variation. The southern and Northern-Sudanian benefit of the lowest precipitations and are hence, the most prone to drought, widely reported as one of the main factors hindering KG production^49^. In the context of an inevitable agricultural reduction in these agroclimatic zones, the earlier the mitigation actions are taken, the more successful will be the collection and conservation efforts of KG’s resources.

On the other hand, future environmental conditions remain, even become more favorable to the Pop1 cultivation (with an increase in suitable areas of 588.812% compared to the present day). However, under the severe environmental conditions (RCP8.5), the cultivable area of this genetic Pop1 is predicted to decline from RCP4.5 (− 89,802.711 km^2^) in the Northern and Southern-Guinean zones of Ghana, Benin and Togo. With regards to these results, significant headway can be made by creating more favorable policy environments. Two strategies proposed by Ramirez-Cabral, Kumar and Taylor^23^ can be used to mitigate loss in KG resources and diversity: first, the use of all the resources of Pop2 in regions where the stress of climate conditions become unsuitable for the species production. An example of this is the Southern-Sudanian zone of Central Benin, where the use of KG is more important than other regions. The second option was to address future loss of suitability by shifting Pop2's current cultivation areas to new regions, such as the Northern-Guinean zone of Southern Benin, Ghana and Togo where future climatic conditions are projected to become more cultivable for KG.

### Conservation implications

Successful management of an endangered species often depends on the accurate identification of current and future cultivable areas. Thus, we combined genetic diversity and ecological niche modeling to understand the evolutionary dynamics in Kersting’s groundnut species across its cultivated regions. Although the predictive models showed that future climatic conditions will be more favorable to KG production, the Sudanian and some areas of Northern-Guinean zones were identified as climatically unsuitable for the species. Notwithstanding the above results, this approach may provide a valuable tool for genetic resources managers for implementing collection and conservation strategies of this orphan legume, as sustained by Sohn et al.^50^ in their study on the endemic bird in South America. In particular, our suggestion is to focus on the Pop2 with particularly high conservation value. This genetic group is represents the genetic diversity including all the coloured Landraces grown by farmers, which is unvaluable resources for the plant genetic improvement. These resources are still managed by farmers through an informal seed system. However, the local seed system alone will likely be insufficient to adapt the crop production to changing climate. In doing so, the formal seeds management efforts can improve the decision-making process that would greatly help in the collection and conservation of the genetic Pop1 at higher risk of extinction but also the Pop1 predicted to thrive with climate change. In general, African crop genetic resources conservation is unfortunately poorly supported by National Governments, and material from the regions is not fully represented in the important international genebanks which afford the baseline for sustained public breeding efforts^45^. In many areas where KG is produced, its production is declining gradually and consequently followed by the lost in genetic resources. Hence, many of these regions would stand to benefit from the genetic resources of other areas of cultivation, if these resources can be effectively managed and shared. Perhaps more importantly, the international cooperation on genetic resources conservation and use, between countries where KG is produced, will be crucial in adapting the crop to the imminent threats of climate change.

### Implications of ENM results for selection for adaptation

Undertaking any global initiatives to overcome food insecurity challenges requires an assessment of staple crop productivity, quality, and environmentally suitable areas under climate change^23^. KG is a staple food crop and a source of proteins and nutrients for smallholder farmers in West Africa. In this research, we expected to identify the intraspecific genetic group that would be resilient under future climate scenarios, and which areas are concerned by changes in KG diversity. The model projections indicate that a shift in Kersting’s groundnut productive areas is slightly and likely with a loss of cultivability of the Pop2 cultivation areas and an increase for Pop1. Although KG is predicted to gain in a suitable area in future environmental changes, its cultivation is, however, decreasing year to year from its cultivated areas^5,51^. Therefore, it will be crucial to adapt *M. geocarpum* species to the increasingly challenging environmental conditions through the development of new resilient cultivars that meet farmers’ needs. The Pop2 comprises all Landraces (diversity, mainly the coloured landraces) of the species and has been reported having the best phenotypic performance^42^. The individuals in this genetic population can serve as a potential source for diversity on which breeding efforts could be based to confer best performance for plant growth and yield to Pop1, mainly composed of the white landrace mostly preferred, grown, and sold by farmers in Benin^5,6^. Similarly, the genetic Pop1 can serve as potential parents to confer resilience to changing climates and increase sustainabilityof genetic Pop2. In addition, advancements in molecular plant breeding would be an importance of paramount to increase the genetic gains and make more accurate the breeding process. In the particular case of KG, currently, available partial GBS data^16,34^ would allow the analyses of intraspecific genetic clusters based on gene network variation for various important phenotypic traits (e.g.: grain yield, yield-related traits, flowering time). This novel approach would provide possibilities to assess the extent to which key functional genes and genetic variation may be threatened under future ecological conditions^52^. Plant breeders have widely and successfully implemented genome-wide breeding approach for the development of climate-resilient varieties^53–55^, through marker-assisted selection and genomic selection. Another approach is to increase variability within the species, particularly in Pop1, clustering mostly white seed coloured individuals, through mutation methods (using physical or chemical mutagens) combined with molecular markers (Targeted Induced Local Lesions in Genomes (TILLING)). Such techniques have been successfully used in breeding of many legume crops to enhance diversity and to develop mutant cultivars^2,56,57^.

## Conclusion and perspectives

This study assessed the potential impacts of climate variations on environments suitability for Kersting’s groundnut cultivation, and consequently its distribution around four West-African countries. The use of Maxent’ models and genetic information allowed a preliminary understanding of the stress factors influencing the climate suitability of the species and genetic populations under two future scenarios (2055, RCP4.5 and RCP8.5). The overall trend shown by our results indicates an increase in climate suitability for the species cultivation in the Northern-Guinean zone of Southern Benin and Togo. However, an important decrease was predicted in other agroclimatic areas, while genetic Pop1 production areas increase. Our findings illustrate also that projected areas of environments cultivability for *M. geocarpum* and the two populations are on different climate change trajectories. The projected distribution maps presented in this paper have been hence, analysed and used to identify strategic measures to manage the impacts of reduced climate cultivability while taking advantage of the opportunities in areas of improved suitability for Kersting’s groundnut cultivation in the future. Our findings could be downscaled to a country level to assist national policymakers in developing strategic control initiatives to prevent the scarcity of this legume.

Although this study represents the first insight into examining the potential of Kersting’s groundnut as a resilient crop under climate change, several limitations are to be noted to develop the right tools to reduce model uncertainty and make better predictions in future research. First, the global distribution of the species and its wild relatives is still incompletely documented online: collecting more and finer occurrence data, especially in regions where its production was previously reported would greatly help in refining or confirming our results. Second, our models identified rainfall, temperature, and soil variables that contributed significantly to their fits. However, socioeconomic factors such as the local market value of the species and cultural preferences must also be considered in the predictions. Combining also measures of key phenotypic traits and botic stress factors (pests ans diseases associated with KG production) in the modeling process would contribute to improving predictions of the impact of climate change on this legume crop. Third, the non-availability of whole genome-wide data in the case of Kersting’s groundnut limited access to phylogenetic information and identification of key functional genes for various important phenotypic traits. That may provide means to assess the response of key functional genes under evolutionary climate change.

Nevertheless, using our approach, we identified species, genetic populations, and cultivable areas for further germplasm collecting to enhance available germplasm and better direct Kersting’s groundnut breeding priorities in the future.

## Materials and methods

### Genotyping and genetic clustering

For the present work, a total of 361 accessions of KG collected from Benin, Burkina Faso, Ghana and Togo were used. The DNA of each sample was extracted from young leaves of each accession, using the protocol of the Integrated genotyping service and support (IGSS) at the Biosciences Eastern and Central Africa (BecA: http://hub.africabiosciences.org/activities/services) located in Nairobi, Kenya. The quality of the DNA was confirmed by electrophoresis in 0.8% agarose, and the quantification was carried out using UVP BioDoc-It2 Imaging System. All of the samples were diluted to 50 ng/µl for the DArT genotyping platform. Genotyping was performed using the DArT-Seq™ platform at Diversity Arrays Technology^58^. The quality analysis of the genotypic data was performed using Illumina HiSeq 2500^59^. The SilicoDArt calling algorithms (DArTsoft14) were used to score DArTseq markers into a binary format (presence = 1 and absence = 0) for each sample genomic representation. A total of 2844 SNP markers were obtained and processed in TASSEL v5^60^, for quality check. SNPs were filtered with TASSEL v5 for further analysis using the proportion of missing data < 20%. A total of 2323 SNPs were retained and the Nipals model in kdcompute (https://kdcompute.igss-africa.org) was used for data imputation.

The program Structure 2.3.4^61^ was used to assign individuals to different genetic clusters based on the admixture model. The population structure was performed based on the Bayesian clustering approach using the following settings: correlated allelic frequencies, burn-in period of 20,000 and 20,000 Markov Chain Monte Carlo (MCMC) interactions; and grouping (K) ranging from 1 to 5 in 10 independent runs. The results generated were used as input into the POPHELPER version 2.3.1, an R package^62^, to determine the most likely genetic clusters based on the Evanno method^63^ and to generate the delta K (ΔK) graph and bar graph. The most likely number of K clusters was determined by its higher ΔK value. After these searches, we found a likely number of K = 2.

The molecular variation (AMOVA) was determined using the R package adegenet^64^ to test for statistically significant differences between the two genetic groups. The pairwise Fst comparisons between the two groups were calculated with 9999 permutations using package ‘hierfstat’ v 0.5–10 in R environment. General patterns of genetic diversity were also evaluated by calculating observed (Ho) versus expected (He) heterozygosity and gene diversity (Hs) within each population. Based on the genetic analyses, we classified the two clusters as distinct genetic populations (Pop1 and Pop2) encompassed within three agroclimatic zones of Burkina Faso, Benin, Ghana, and Togo (northern-Sudanian, southern-Sudanian, and northern-Guinean). We then used these genetically defined populations and their GPS points available, to define populations' location points.

### Occurrence data

The occurrence data for *M. geocarpum* was obtained from the Global Biodiversity Information Facility (GBIF, www.gbif.org), an online available database, and the crop self-collected material of the Laboratory of Genetics, Biotechnology, and Seeds Science (GBioS). As the different data sources and a large dataset (> 500 occurrence records) would likely carry elevated geographical or environmental space biases^65,66^, the number of records were decreased in Wallace package, an online workspace based on R interface^67^ using four complementary approaches: (1) we first removed occurrences collected before 1986 to match with environmental layers and soil properties; (2) considerable ambiguity may exist in GBIF data over the identity of the species due to synonymous names (*M. geocarpum* var. *geocarpum*, *M. geocarpum* var. *Tisserantii*; *Kerstingiella geocarpa*, *Kerstingiella tisserantii*). To avoid any confusion arising from this taxonomic ambiguity, we searched through the online databases using the following keywords: *Macrotyloma geocarpum*, *Kerstingiella geocarpa*, var. *geocarpa*, or var. *geocarpum*; orphan legumes. We then harmonized the GBIF database and discarded the reports on var. *tisserantii* and *Kerstingiella tisserantii*; (3) we used spatially filtering occurrences located ≤ 10 km from other occurrences using the spThin^68^, an R package; finally, (4) we manually checked isolated locations points in Africa (in ArcGIS ver. 10.7.1) and removed occurrences in areas where *M. geocarpum* is not generally grown.

The defined genetic clusters data with their geographic coordinates were also filtered separately to ensure the real distribution of each population within agroclimatic zones. After data cleaning steps, the final occurrence datasets contained: for the species, a dataset of 53 occurences (which include 17 points retained from GBIF), used for modeling without genetic information. And two data files that comprised 24 locations points for Pop1, and 26 occurences for Pop2, used in subsequent analyses integrating genetic information (see Supplemental Table S3).

### Environmental variables

We used bioclimatic layers combined with soil properties to project current and future niches for the species and each genetic group. A total of 15 bioclimatic variables were downloaded from Africlim online regional climate models (RCMs) data portal (https://webfiles.york.ac.uk/KITE/AfriClim/)^69^. Current and future variables averaged between the periods 1986–2015 (2000) and 2041–2060 (2055) were downloaded at a 30 arc-s (~ 1 km) spatial resolution. For future climatic conditions, predictions from the Ensemble model^69^ were used. This model simulates changes based on a set of scenarios. The projections were run under Representative Concentration Pathway (RCP), RCP 4.5, and RCP 8.5 for the 2055 time horizon^70^. In all RCPs, the climatic conditions are extreme in RCP 8.5 scenarios compared to RCP 4.5. RCP 4.5 projects temperatures to rise above industrial levels by at least 1.5 °C in West Africa, with atmospheric CO_2_ reaching 500 ppm while in RCP 8.5 projections, temperatures are predicted to rise by 2.8 °C and atmospheric CO_2_ to be over 550 ppm^71^. These climate projections were statistically downscaled to match the bioclimatic variables using the delta method^72^.

Data related to soil characteristics were available in the World Soil Information (ISRIC) databases (Soil-property-maps-of-Africa-at-250-m-resolution) at 250 m resolution^73^. These spatial predictions of soil properties were generated based on two predictive approaches such as random forests and linear regression^73^. Soil characteristics included 30 variables related to the soil's physical, chemical, and nutritional properties. Soil data were then converted to 30 arcseconds using ArcGIS software v 10.7.1 to match with bioclimate layers. Finally, using shapefile boundaries of four West African countries (Benin, Burkina Faso, Ghana, and Togo) we cropped all variables to encompass the broad geographic regions that define Kersting’s groundnut global distribution.

We discarded highly correlated variables (≥ 0.8) by Pearson´s rank correlation coefficients analysis using the R library ‘ntbox’^74^(see correlation matrix in Suplementary Table S4). A total of 18 variables were retained as less correlated. We then conducted a few preliminary model runs without genetic information (with species occurrences) in Maxent v. 3.4.4^75^ to reduce the number of variables to be included in the prediction models^75^. For each run, we removed variables with the lowest contribution. Six bioclimatic variables including Max Temperature warmest month (bio5), Mean annual rainfall (bio12), Rainfall driest month (bio14), Bulk density in kg/cubic-meter for 10 cm depth (blt_d2), soil texture fraction clay at 10 cm depth (clyppt_d2), = Soil texture fraction silt in percent for 10 cm depth (Sltppt_d2) were chosen based on their noncollinearity and contribution to the prediction models and were used in the final models of the species, and with genetic information (Pop1 and Pop2).

### Ecological predictive models’ development and evaluation

To calibrate our models, we employed the maximum entropy method^75,76^ implemented in Maxent ver. 3.4.4^75^. The algorithm has been extensively tested and benchmarked^77,78^. Many studies have reported Maxent as one of the highest performing presence-background algorithms^21,79^. As the selection of sample points can influence model performance in Maxent^80^, we restricted the selection of background points using the regularization of 10,000 background points^21^. Models were trained with data from the present and projected in the future. Three Maxent models were generated with different occurrence datasets:

- model 1: all accessions location points together without genetic information were used in projecting the entire distribution of KG, distributed in four West African countries (Burkina Faso, Benin, Ghana, and Togo) where the distribution range of the species is characterized by three agroclimatic zones including the Northern-Sudanian, Southern-Sudanian, and Northern-Guinean.
- model 2 and model 3: were developed using occurrences of Pop1 and Pop2, respectively the genetically defined populations. Pop1 location points are covered by the agroclimatic zones of Southern-Sudanian, and Northern-Guinean of Southern and central Benin, and Northern Togo. On the other hand, the occupation range of Pop2 extends over the four countries and the three agroclimatic zones.

We used a Bootsrap method, by defining 75% of the data for model training and 25% for model testing for 10 iterations^76^. We Kept the Auto Features box checked Linear, quadratic, product, hinge, and threshold functions of predictor variables were employed, and variable importance was assessed using a jackknife analysis^79^. For easy interpretation of the results of niche models, we specified the Maxent’ output format to a logistic form.

The three models performance was tested based on the partial receiver operator characteristics (pROC) and the corrected Akaike information criterion (AICc) approaches. We used the outputs from Maxent to perform pROC in R package ‘ENMGadgets’ v 0.1.0.1^81^, by specifying 500 iterations with the omission threshold set at five percent. The statistical significance of pROC values was examined using t-test statistic. The AICc was estimated based on the number of parameters and likelihoods of continuous raw outputs, using R library ENMEval v 2.0^82^, following the method described by Warren and Seifert^83^. The lower is the value of AICc, more accurate is the predictive model^84^. Models omission rates calculated with the 10th percentile training presence threshold, and the area under the receiver operating characteristic curve (AUC), were also used as secondary criteria for testing performance of each model^66,85^. A model is considered as having a good fit when its AUC is closed to one (AUC ≥ 0.75)^21^. The final models were projected onto future climatic scenarios (RCP4.5. and RCP8.5) of the horizon 2055, using Maxent software with ten bootstrap iterations. Each model was set to thresholds with the 10 percentile training presence values to produce continuous and binary raster maps for current and future scenarios in ArcGIS v. 10.7.1.

Two different levels were, therefore, defined: unsuitable and suitable. Finally, we quantified in km^2^ the surface occupied by each condition (suitable and unsuitable) using the continuous raw outputs from maxent in ArcGIS v. 10.7.1. We estimated the range size change under scenarios RCP 4.5 and RCP 8.5 of the period 2055 for the species and two genetic populations, following the model used by Hu et al.^86^:

$$\Delta \left(\%\right)=\frac{\left(FA-CA\right)}{CA}\times 100$$

where, FA corresponds to the future suitable areas (in km^2^) under a given future scenario; CA is the suitable areas in current conditions. Negative, null, and positive values represent range lost, stable, and gained, respectively. Furthermore, to visualize the potential changes of suitable areas for Kersting’s groundnut production, we compared current and future distribution ranges of the crop and genetic populations using package “tmap” version 3.3-1^87^ in R. Finally, to determine the zones where each model transfers would require extrapolation under future climatic conditions, we used the mobility-oriented parity (MOP) metric. The MOP analysis was performed with the R library ‘ntbox’ by setting for environmental distances to the nearest 10% of the reference region and 500 runs.

### Niche differentiation

To understand whether differences between species and genetic populations emerge from true niche divergences, we performed Schoener’s niche equivalency (identity) and similarity test (D), and Warren’s niche background test (I) using the R package ‘humboldt’^35^. Niche equivalency test is used to test out the null hypothesis that two species have identical environmental niches. The niche equivalency test examines the observed niche similarities between the predicting models of two species. The niche background test evaluates the power to detect differences between two ENMs. Compared to the existing tests, these methods improve the accuracy of niche similarity quantifications and corresponding statistical tests^35^. The Schoener's D statistic is used to determine how similar the occupied niches of two species are based on the original input occurrences. The observed values of D in both the niche identity test and the background similarity test were compared to the mean values of the randomized runs using t-test^88^. Hence, the environmental niches were considered significantly different if the observed values of niche overlap were less than 95% or 99% (alpha = 0.05 and 0.01, respectively) of the overlap values derived from the pseudoreplicates.

We visualized and analyzed E-space as two axes of a Principal Component Analysis (PCA) of input environmental variables throughout the entire study areas of the species and genetic populations. Broennimann et al.^89^ used a kernel density function^90^ to create a continuous E-space surface in a grid of 100 × 100 cells, estimating the occupied E-space of the focal species or its environment, respectively, using PC values from either the input occurrence localities or study region data.

Furthermore, The species fundamental niche from observed niche was analyzed with the packge ‘humboldt’^35^. The fundamental niche was characterised by truncating species occupied E-space by the available E-space in its environment. The larger the truncated proportion, the greater the risk that the occupied niche does not accurately reflect fundamental niche of the species. Hence, the Potential Niche Truncation Index (PNTI) was estimated to measure the amount of the observed E-space truncated by the available E-space. When the values of PNTI are ranged between 0.15 and 0.3, there is a moderate truncation risk while values > 0.3 explained a high risk of niche truncation due to limited available E-space^35^.

## Supplementary Information

Supplementary Information.

## Data Availability

The molecular data underlying this article (DarTSeq data) will be submitted to NCBI.
