# Supplementary material for: Coupling genetic structure analysis and ecological-niche modeling in Kersting’s groundnut in West Africa
Source: Sci Rep. 2022 Apr 4;12:5590. doi: 10.1038/s41598-022-09153-5 (PMC8980027; doi:10.1038/s41598-022-09153-5)
Supplement: Supplementary file 1 — Supplementary Information. [file 41598_2022_9153_MOESM1_ESM.pdf]

Table S1

loci	ind	k	runs	elpdmean	elpdsd	elpdmin	elpdmax	lnk1	lnk1max	lnk1min	lnk2	lnk2max	lnk2min	deltaK
**2323**	361	1	10	-46877.69	5.43270957564781	-46883.1227095756	-46872.2572904244	NA	NA	NA	NA	NA	NA	NA
**2323**	361	2	10	-33188.93	113.317401331148	-33302.2474013312	-33075.6125986688	13688.76	13796.645	13580.875	11549.02	11596.905	11501.135	101.917
**2323**	361	3	10	-31049.19	173.317867579261	-31222.5078675793	-30875.8721324207	2139.74	2199.74	2079.74	1440.19	2315.58	564.8	8.31
**2323**	361	4	10	-30349.64	1108.70749594091	-31458.3474959409	-29240.9325040591	699.55	1634.94	-235.84	1128.78	1834.299	423.261	1.018
**2323**	361	5	10	-30778.87	878.836503629152	-31657.7065036291	-29900.0334963708	-429.23	-199.359	-659.101	NA	NA	NA	NA

Table S2

Species	Period	Suitable area (km2)	Total area used (km2)	Suitable area (%)
**M. geocarpum**	Current	148383.56	687667.214	21.578
	RCP4.5	283615.994	687654.638	41.244
	RCP8.5	172967.017	687662.393	25.153
**Pop1**	Current	16662.979	687704.399	2.423
	RCP4.5	114776.675	687663.846	16.691
	RCP8.5	24973.963	687709.305	3.631
**Pop2**	Current	162948.942	687666.565	23.696
	RCP4.5	55796.456	687690.084	8.114
	RCP8.5	37574.765	687701.734	5.464

Table S3

Name	Longitude	Latitude	Elevation	Climatic zone	Locality	Country	Year	SCC	Acc
**Mg_Pop1**	-4.290194	11.170056	458	Southern-Sudanian	Bobo Dioulasso	Burkina	2018	White	MC002
**Mg_Pop1**	0.550003	10.75	177	Southern-Sudanian	Natongou	Togo	2016	Black	AF227
**Mg_Pop1**	1.109833	10.348472	220	Southern-Sudanian	Manta	Benin	2016	White	AF021
**Mg_Pop1**	1.680889	7.58325	217	Southern-Sudanian	Koutoukpé	Benin	2016	White	AF050
**Mg_Pop1**	1.750722	7.410389	154	Southern-Sudanian	Gounoukouin	Benin	2016	White	AF027
**Mg_Pop1**	1.837111	7.329389	143	Southern-Sudanian	Sovlegni	Benin	2016	White	AF060
**Mg_Pop1**	1.965944	7.137222	196	Northern-Guinean	Dékanmey	Benin	2016	White	AF075
**Mg_Pop1**	2.025917	7.229639	231	Southern-Sudanian	Adamè	Benin	2016	White	AF011
**Mg_Pop1**	2.076556	7.085111	86	Northern-Guinean	Attia	Benin	2016	White	AF010
**Mg_Pop1**	2.130944	7.23375	167	Northern-Guinean	Zouzonmè	Benin	2016	White	AF236
**Mg_Pop1**	2.145611	7.708778	114	Southern-Sudanian	Fita	Benin	2016	White	AF114
**Mg_Pop1**	2.160333	8.443806	263	Southern-Sudanian	Soladji Sowignandji	Benin	2016	White	AF119
**Mg_Pop1**	2.226472	8.262278	216	Southern-Sudanian	Aklamkpa	Benin	2016	White	AF229
**Mg_Pop1**	2.23875	7.223028	49	Northern-Guinean	Za-Kékéré	Benin	2016	Black	AF299
**Mg_Pop1**	2.293722	7.988083	183	Southern-Sudanian	Agouagon	Benin	2016	White	AF127
**Mg_Pop1**	2.324	7.355611	136	Northern-Guinean	Adogo	Benin	2016	White	AF001
**Mg_Pop1**	2.393528	7.640861	102	Southern-Sudanian	Gbèdolo-N'Gbèga	Benin	2016	White	AF095
**Mg_Pop1**	2.420806	7.762278	124	Southern-Sudanian	Bêtêkoukou	Benin	2016	White	AF084
**Mg_Pop1**	2.434056	7.475667	58	Northern-Guinean	Igbohounla	Benin	2016	White	AF006
**Mg_Pop1**	2.616639	8.165944	245	Southern-Sudanian	Dègué-Dègué	Benin	2016	White	AF148
**Mg_Pop1**	2.623278	8.040694	176	Southern-Sudanian	Agatoudji	Benin	2016	White	AF192
**Mg_Pop1**	2.710528	8.158222	160	Southern-Sudanian	Atta	Benin	2016	White	AF151
**Mg_Pop2**	1.05	9.933336	280	Southern-Sudanian	Atétou	Togo	2016	Black	AF211
**Mg_Pop2**	1.052083	10.106861	208	Southern-Sudanian	Pimini	Togo	2016	Black	AF203
**Mg_Pop2**	-4.923861	11.00975	545	Southern-Sudanian	Nialé-Salé	Burkina	2018	White_black_eye	MC010
**Mg_Pop2**	-4.840056	11.981056	320	Southern-Sudanian	Toussianmasso	Burkina	2018	White_black_eye	MC013
**Mg_Pop2**	-4.77525	11.614667	352	Southern-Sudanian	Gangarla	Burkina	2018	Black	MC016
**Mg_Pop2**	-4.735167	11.939139	334	Southern-Sudanian	Djêouiya (Kourinion C)	Burkina	2018	White_black_eye	MC011
**Mg_Pop2**	-4.513083	10.981167	541	Southern-Sudanian	Tien M'Bèya	Burkina	2018	White_black_eye	MC009
**Mg_Pop2**	-4.430167	11.133083	379	Southern-Sudanian	Koumi	Burkina	2018	Black	MC008
**Mg_Pop2**	-4.290194	11.170056	458	Southern-Sudanian	Témékina	Burkina	2018	Black	MC027
**Mg_Pop2**	-3.708778	11.963278	363	Northern-Sudanian	Kéra	Burkina	2018	Black	MC018
**Mg_Pop2**	-3.680083	12.188222	279	Northern-Sudanian	Kékaba	Burkina	2018	Black	MC023
**Mg_Pop2**	-3.674444	12.071278	327	Northern-Sudanian	Syn	Burkina	2018	Black	MC026
**Mg_Pop2**	-3.583222	12.192528	308	Northern-Sudanian	Poundou	Burkina	2018	White_black_eye	MC021
**Mg_Pop2**	-2.700083	10.532444	307	Southern-Sudanian	Nimbare	Ghana	2018	Brown _greyed_orange_eye	MC055
**Mg_Pop2**	-2.665111	10.646	292	Southern-Sudanian	Die	Ghana	2018	Brown _greyed_orange_eye	MC078
**Mg_Pop2**	-2.594278	10.487778	285	Southern-Sudanian	Sanwie	Ghana	2018	Brown _greyed_orange_eye	MC047
**Mg_Pop2**	-2.258972	10.566222	238	Southern-Sudanian	Setuori	Ghana	2018	Brown _greyed_orange_eye	MC088
**Mg_Pop2**	1.078639	10.137778	231	Southern-Sudanian	Nadoba	Togo	2016	Black	AF224
**Mg_Pop2**	1.206889	10.185722	564	Southern-Sudanian	Koussoucoingou	Benin	2016	Red	AF196
**Mg_Pop2**	1.471444	10.094389	374	Southern-Sudanian	Pam-Pam	Benin	2016	White_black_eye	AF200
**Mg_Pop2**	1.965944	7.137222	196	Northern-Guinean	Dékanmey	Benin	2016	Black	AF077
**Mg_Pop2**	2.025333	7.229444	243	Southern-Sudanian	Adamè	Benin	2016	Red	AF067
**Mg_Pop2**	2.178306	7.257	150	Northern-Guinean	Djoyitain	Benin	2016	Red	AF265
**Mg_Pop2**	2.279611	7.205639	41	Northern-Guinean	Kèmondji	Benin	2016	Red	AF254
**Mg_Pop2**	2.660333	8.10975	204	Southern-Sudanian	Igboloko	Benin	2016	White	AF157
**Mg_GBIF**	-2.66667	10.83333	282	Southern-Sudanian	Navrongo	Ghana	1982		
**Mg_GBIF**	-2.66242	10.36713	273	Southern-Sudanian	Nadowli	Ghana	1998		
**Mg_GBIF**	-2.5387	10.46414	324	Southern-Sudanian	Dafiama	Ghana	1994		

**Mg_GBIF**	-2.43029	9.24525	325	Southern-Sudanian	Sawla	Ghana	2007		
**Mg_GBIF**	-1.91667	9.16667	226	Southern-Sudanian	Jimbale	Ghana	1982		
**Mg_GBIF**	-1.46667	10.41667	190	Southern-Sudanian	Lambussi	Ghana	1982		
**Mg_GBIF**	0.2	10.55	176	Northern-Sudanian	Dapaong	Togo	1983		
**Mg_GBIF**	0.3	10.73333	329	Northern-Sudanian	Dapaong	Togo	1983		
**Mg_GBIF**	0.58333	10.75	149	Northern-Sudanian	Dapaong	Togo	1983		
**Mg_GBIF**	1.1	9.8	480	Southern-Sudanian	Niamtougou	Togo	1983		
**Mg_GBIF**	1.28333	7.8	176	Northern-Guinea	Elavagnon	Togo	1984		
**Mg_GBIF**	1.33333	9.75	410	Southern-Sudanian	Kara	Togo	1983		
**Mg_GBIF**	1.9333	7.3333	159	Northern-Guinea	Djidja	Benin	2013		
**Mg_GBIF**	1.94	7.42333	151	Northern-Guinea	Djidja	Benin	2001		
**Mg_GBIF**	2.3	7.9833	196	Southern-Sudanian	Agouagon	Benin	2013		
**Mg_GBIF**	2.35	6.45	18	Northern-Guinea	Abomey-Calavi	Benin	2013		
**Mg_GBIF**	-2.546625	10.015748	323	Southern-Sudanian	Nakori	Ghana	1999		

Table S4

var_names	bio1	bio10	bio11	bio12	bio13	bio14	bio15
bio1	1	0.844355830184557	0.927433364995828	-0.499383375120073	-0.00273090791745665	-0.251746409760211	-0.0453067110298509
bio10	0.844355830184557	1	0.704548881586976	-0.725676769697563	0.397195220886749	-0.597356483783441	0.383036591710093
bio11	0.927433364995828	0.704548881586976	1	-0.486986437491026	-0.108766939535194	-0.0518400282045932	-0.223918538924989
bio12	-0.499383375120073	-0.725676769697563	-0.486986437491026	1	-0.280914986558759	0.504064572866001	-0.167185586982284
bio13	-0.00273090791745665	0.397195220886749	-0.108766939535194	-0.280914986558759	1	-0.507761268027991	0.944100441958632
bio14	-0.251746409760211	-0.597356483783441	-0.0518400282045932	0.504064572866001	-0.507761268027991	1	-0.60267234607214
bio15	-0.0453067110298509	0.383036591710093	-0.223918538924989	-0.167185586982284	0.944100441958632	-0.60267234607214	1
bio16	-0.111169539650991	0.288596618031018	-0.295070741090137	-0.0448772145642807	0.914863853931932	-0.551185238389875	0.988258650269329
bio17	-0.16524960005822	-0.587653219939693	0.0269057639384838	0.502984676484521	-0.71093072344192	0.865282304651458	-0.804389086826283
bio2	0.125573629496233	0.575721425045807	-0.0964272440694396	-0.453220525178487	0.610575824336238	-0.813256329695803	0.759677277738676
bio3	-0.154504893894777	-0.618718879727624	-0.0349163460218751	0.659484280974973	-0.79037577690147	0.644364323518178	-0.74281669361889
bio4	0.266452043955033	0.726711314613807	0.0534769766840484	-0.62009848911058	0.67581331040747	-0.786305192921593	0.733159689844378
bio5	0.630798624058062	0.889273839355155	0.418965126942068	-0.59189374435318	0.432162461279748	-0.778804006653733	0.540266851035058
bio6	0.178010649413025	-0.356606141835318	0.325267231279454	0.460962255944177	-0.744565986740888	0.645769205681607	-0.79100845740166
bio7	0.151993736167648	0.637255137239486	-0.0431863109500993	-0.579827431811806	0.707401945997529	-0.790248022502578	0.787022182327386
blt_d1	0.493214668568933	0.7560326513905	0.358144857863154	-0.68443953829038	0.564625607349778	-0.513200713701101	0.51491261519899
blt_d2	0.537772814043996	0.769701847553339	0.411012922245985	-0.696690191235659	0.506342793324941	-0.494288182492599	0.45179135204703 5
blt_d3	0.576484022277446	0.760388112995033	0.45248258016206	-0.67284648552222	0.415666744253346	-0.463066080330132	0.366991016765645
cec_d1	-0.160302571238014	-0.194353166677858	-0.115988894934154	0.17339435330712	-0.0698916317718166	0.248738336882847	-0.0778712157070734
cec_d2	-0.105499512382095	-0.142345587660525	-0.0715723625755463	0.184441059703355	-0.0570080746338768	0.222509478261147	-0.0460954149519256
cec_d3	-0.0690583257013816	-0.0795285203914554	-0.0464101687224274	0.125712829335954	0.016894308715227	0.171382431250614	0.0115920150305794
clyppt_d1	-0.0725885434113605	0.166391042349618	-0.0703968644866863	-0.301582063727054	0.534703090105895	-0.287746623847822	0.45408765021111 4
clyppt_d2	-0.0934600748178284	0.17439382765816	-0.101802018298466	-0.290563289315818	0.596101949158034	-0.328072621380567	0.526057678507092
clyppt_d3	-0.0649865732015405	0.248003202059693	-0.0887872455657293	-0.355957170522383	0.692090730564467	-0.398843965596179	0.618352330610155
eackcl_d1	-0.425729922464546	-0.287156642056327	-0.400093354077002	0.332046463873509	0.334172445691002	-0.00812986327651255	0.352971798697582
eackcl_d2	-0.421663370842963	-0.277773248218127	-0.400068993641938	0.328475150015816	0.313517717208234	-0.0273155532108618	0.344618844875416
eackcl_d3	-0.424479731573192	-0.276626124361032	-0.397073488271266	0.330641033829471	0.333868413397336	-0.0292019093116052	0.360042088672109
ecax_d1	0.0799685646874823	-0.0826964717185411	0.199585615378019	-0.155982264703853	-0.295224048712725	0.163374947098307	-0.453166486987061
ecax_d2	0.146030019156351	0.0655644089843809	0.109655922349013	0.0456360322674223	-0.00538958242778555	-0.028120328258814	-0.0237707937060184
emgx_d1	0.354187641919206	0.212435127031584	0.398900642883212	-0.158497251230984	-0.123366058393418	0.105723096530503	-0.253880064048325
emgx_d2	0.344658909961484	0.214163833319058	0.383590673142573	-0.174455007570175	-0.128015140306531	0.0817175876343479	-0.243860573382503
exkx_d1	-0.139341089233197	-0.341475842503002	-0.0442309200125858	0.194654169402331	-0.424908974669841	0.430057777106955	-0.489378147652451
exkx_d2	-0.16282880618948	-0.323498508048356	-0.0633469817146399	0.133887941716703	-0.331237339433273	0.385297633349865	-0.400470977897709
orcdrc_d1	-0.158563655681715	-0.494188598949919	-0.0254384636412572	0.343024686527785	-0.621920787546661	0.600421174910789	-0.698962601092968
orcdrc_d2	-0.0786693683552278	-0.327809538611576	0.0146497488602977	0.188145775451055	-0.391559032675994	0.360848494300371	-0.491375019986821
orcdrc_d3	-0.0927606969642114	-0.308036307798098	0.0343756228210862	0.107467843456584	-0.29053681065566	0.407771201223569	-0.434121028017781
phihox_d1	0.28887966669154	0.0855104531340121	0.301546969096923	-0.0384780420212017	-0.437031281047383	0.0408101739592995	-0.390295794187404
phihox_d2	0.373557805044893	0.195221730283765	0.353042527192001	-0.122520977527948	-0.3571712281156	-0.0546194593365677	-0.322148610404853
phihox_d3	0.341499184581521	0.156274824335811	0.343849576406944	-0.0849514347512398	-0.370073037201218	0.0313155634490795	-0.35163828708426
sltppt_d1	0.232008636646802	0.563591686679406	0.128332870556178	-0.610481796326491	0.620752721987418	-0.551866243223664	0.567888460007792
sltppt_d2	0.212294467697783	0.548653117418333	0.116238801724913	-0.657879226773966	0.612354437788979	-0.563087819010142	0.546168202641261
sltppt_d3	0.192059196662323	0.532018923963168	0.0983012081326863	-0.624652236972495	0.641601366003185	-0.54557614389598	0.57013567379532
sndppt_d1	-0.0957497662124756	-0.404691030106469	-0.0434699217157918	0.52810469018415	-0.624963151424098	0.474119657688967	-0.550298561388372
sndppt_d2	-0.0517204112538598	-0.382518957544408	0.00296171282780853	0.505361812881634	-0.66513385349088	0.484655089414679	-0.591799419504989
sndppt_d3	-0.0660230528948089	-0.415703221753498	-0.000458167930197022	0.516760767956027	-0.720265269971371	0.512402046868939	-0.644318942829863

var_names	bio16	bio17	bio2	bio3	bio4	bio5	bio6	bio7
bio1	-0.111169539650991	-0.16524960005822	0.125573629496233	-0.154504893894777	0.266452043955033	0.630798624058062	0.178010649413025	0.151993736167648
bio10	0.288596618031018	-0.587653219939693	0.575721425045807	-0.618718879727624	0.726711314613807	0.889273839355155	-0.356606141835318	0.637255137239486
bio11	-0.295070741090137	0.0269057639384838	-0.0964272440694396	-0.0349163460218751	0.0534769766840484	0.418965126942068	0.325267231279454	-0.0431863109500993
bio12	-0.0448772145642807	0.502984676484521	-0.453220525178487	0.659484280974973	-0.62009848911058	-0.59189374435318	0.460962255944177	-0.579827431811806
bio13	0.914863853931932	-0.71093072344192	0.610575824336238	-0.79037577690147	0.67581331040747	0.432162461279748	-0.744565986740888	0.707401945997529
bio14	-0.551185238389875	0.865282304651458	-0.813256329695803	0.644364323518178	-0.786305192921593	-0.778804006653733	0.645769205681607	-0.790248022502578
bio15	0.988258650269329	-0.804389086826283	0.759677277738676	-0.74281669361889	0.733159689844378	0.540266851035058	-0.79100845740166	0.787022182327386
bio16	1	-0.749279228524652	0.712148713141521	-0.66129284545187	0.656180752169187	0.470707901254828	-0.738366758459705	0.719946628928502
bio17	-0.749279228524652	1	-0.920392885138716	0.782927155224612	-0.824659797240843	-0.80895439630542	0.800365637275814	-0.911066112277242
bio2	0.712148713141521	-0.920392885138716	1	-0.749446478909404	0.88806752233027	0.831482368185797	-0.862355669610569	0.964077633041928
bio3	-0.66129284545187	0.782927155224612	-0.749446478909404	1	-0.869775014988513	-0.639515997929546	0.883168899951459	-0.894597701291979
bio4	0.656180752169187	-0.824659797240843	0.88806752233027	-0.869775014988513	1	0.815309169323088	-0.845804712610805	0.945480402892309
bio5	0.470707901254828	-0.80895439630542	0.831482368185797	-0.639515997929546	0.815309169323088	1	-0.531918890271584	0.807741386662521
bio6	-0.738366758459705	0.800365637275814	-0.862355669610569	0.883168899951459	-0.845804712610805	-0.531918890271584	1	-0.928870051824457
bio7	0.719946628928502	-0.911066112277242	0.964077633041928	-0.894597701291979	0.945480402892309	0.807741386662521	-0.928870051824457	1
blt_d1	0.448602950612171	-0.547532449990338	0.57768016286954	-0.719257123215272	0.75572016123453	0.675863474563473	-0.552917127920233	0.680576300529822
blt_d2	0.384868212505539	-0.503549941776979	0.539626686057041	-0.679364594005807	0.724144291315	0.671597677479998	-0.498112652321224	0.640555600626177
blt_d3	0.302087778450658	-0.441019787314524	0.491187788247322	-0.603539476318788	0.669295900619896	0.657922606409146	-0.419357979568814	0.579745016747922
cec_d1	-0.0579652149001027	0.210563563374025	-0.170051867428834	0.0606967563823985	-0.167267578729981	-0.219330517466142	0.0552434814623059	-0.134400085980403
cec_d2	-0.0220217730203188	0.17272031371366	-0.14428641890051 2	0.0381228939552023	-0.134563527429365	-0.158904228513119	0.0633296063399375	-0.11359792087319
cec_d3	0.0293744312135818	0.122639398684627	-0.0922598091270205	-0.0340288340708465	-0.0670093027642	-0.109350962650882	0.00128126386902462	-0.0487243828869442
clyppt_d1	0.435760512927589	-0.351112017286208	0.290230725353338	-0.522897841870012	0.347807029047135	0.145959679446742	-0.477500559476945	0.396280589941215
clyppt_d2	0.507131418419285	-0.41588691089752	0.33536414486843	-0.562106326576402	0.383561513091342	0.176590157328717	-0.517498100702652	0.43752519155791
clyppt_d3	0.595774203218544	-0.498828004058587	0.418781988225422	-0.650637113392348	0.477357658941595	0.25314026336462	-0.598866212464643	0.527658084846243
eackcl_d1	0.401988330241274	-0.0578439650559196	0.03857939351178	-0.017994566245785	-0.00795576954173528	-0.178123762011261	-0.144981771617033	0.0230209690535185
eackcl_d2	0.392858046161703	-0.0667619557742531	0.0606274690722593	-0.0170014306454433	0.00741069390517613	-0.155502348929719	-0.152323387247365	0.0380272586369655
eackcl_d3	0.408738525790329	-0.0702517207538805	0.0616769976277098	-0.0251173605113637	0.0104661014725306	-0.157431788718771	-0.158518708737828	0.0414964506479823
ecax_d1	-0.488158891006741	0.394839421931676	-0.469531740359617	0.174943510844535	-0.253211456529905	-0.333547862591875	0.318097303280321	-0.367359349198612
ecax_d2	-0.0117150163073688	0.129961553432005	-0.189476829660698	0.0382647140324277	-0.0133556280740898	-0.0487595031885931	0.171039945361379	-0.140406037584439
emgx_d1	-0.302251243452368	0.260296249392073	-0.337741266392666	0.0190202511194567	-0.0467012373674046	-0.0622085898645183	0.279520294420961	-0.221812719933518
emgx_d2	-0.287339661647386	0.233371769358853	-0.304434353673408	0.021832377107426	-0.0317891665986029	-0.0432868094417676	0.258830964528397	-0.199132077571426
exkx_d1	-0.466159980139231	0.615125375953696	-0.569374418326746	0.419135096461354	-0.403993389184503	-0.519885371454368	0.427080415307583	-0.5247410211296
exkx_d2	-0.378226149788675	0.519559570280437	-0.496624966236669	0.348018212351145	-0.357083402987599	-0.482599515768546	0.344171829903222	-0.450710647736703
orcdrc_d1	-0.676727028525406	0.748380436182492	-0.784390518274887	0.607532926005118	-0.674632247949279	-0.672795464237311	0.666383050779705	-0.758229008684452
orcdrc_d2	-0.488805194722558	0.521859738054821	-0.633623258617535	0.385588349404957	-0.478715449794668	-0.522391573488911	0.495835838585903	-0.573704656079527
orcdrc_d3	-0.425952214881605	0.498121877847377	-0.609938677960685	0.29564293985478	-0.462048569748855	-0.516693402019397	0.431876893532842	-0.526684085071892
phihox_d1	-0.409372586134546	0.128246503529561	-0.150205050354638	0.326864070844836	-0.199215210502576	0.0848205858238864	0.379837973018285	-0.227340062659016
phihox_d2	-0.344720673257443	0.0620011694864595	-0.0657402232925179	0.241633408546787	-0.088347009920418	0.188380953316376	0.311322759270331	-0.134340481641965
phihox_d3	-0.373487390976167	0.124277748789019	-0.141546963637894	0.249080698641329	-0.134789790950369	0.116649135005346	0.342518058753456	-0.187435557750999
sltppt_d1	0.489986616518336	-0.660650856229402	0.620961678415923	-0.81183142705767	0.68612537665873	0.547868128148966	-0.717073792376984	0.73887399113531
sltppt_d2	0.469055060977026	-0.651996258245531	0.617618725419516	-0.811370732426622	0.682664651767945	0.529340233023995	-0.728333365319373	0.738608396339743
sltppt_d3	0.496312238985598	-0.643172501405011	0.607565775198927	-0.816685514479193	0.677233223068292	0.509017584603615	-0.732957490574233	0.732938155684684
sndppt_d1	-0.497851581837056	0.563621078796001	-0.506900632293837	0.723045138870425	-0.561276465983118	-0.389912830395938	0.651360593344032	-0.624031700328379
sndppt_d2	-0.541597121333326	0.582711709932318	-0.515784438028875	0.746721440500032	-0.574963475938009	-0.375879758231839	0.679165903126689	-0.637251338564995
sndppt_d3	-0.59377153084622	0.612898410628796	-0.550469974884735	0.782168770046673	-0.618489720122995	-0.407997609266023	0.712087509921694	-0.674220298351919

blt_d1	blt_d2	blt_d3	cec_d1	cec_d2	cec_d3	clyppt_d1	clyppt_d2	clyppt_d3	eackcl_d1	eackcl_d2	eackcl_d3	ecax_d1	ecax_d2	emgx_d1	emgx_d2
0.493214668568933	0.537772814043996	0.576484022277446	-0.160302571238014	-0.105499512382095	-0.0690583257013816	-0.0725885434113605	-0.0934600748178284	-0.0649865732015405	-0.425729922464546	-0.421663370842963	-0.424479731573192	0.0799685646874823	0.146030019156351	0.354187641919206	0.344658909961484
0.7560326513905	0.769701847553339	0.760388112995033	-0.194353166677858	-0.142345587660525	-0.0795285203914554	0.166391042349618	0.17439382765816	0.248003202059693	-0.287156642056327	-0.277773248218127	-0.276626124361032	-0.0826964717185411	0.0655644089843809	0.212435127031584	0.214163833319058
0.358144857863154	0.411012922245985	0.45248258016206	-0.115988894934154	-0.0715723625755463	-0.0464101687224274	-0.0703968644866863	-0.101802018298466	-0.0887872455657293	-0.400093354077002	-0.400068993641938	-0.397073488271266	0.199585615378019	0.109655922349013	0.398900642883212	0.383590673142573
-0.68443953829038	-0.696690191235659	-0.67284648552222	0.17339435330712	0.184441059703355	0.125712829335954	-0.301582063727054	-0.290563289315818	-0.355957170522383	0.332046463873509	0.328475150015816	0.330641033829471	-0.155982264703853	0.0456360322674223	-0.158497251230984	-0.174455007570175
0.564625607349778	0.506342793324941	0.415666744253346	-0.0698916317718166	-0.0570080746338768	0.016894308715227	0.534703090105895	0.596101949158034	0.692090730564467	0.334172445691002	0.313517717208234	0.333868413397336	-0.295224048712725	-0.00538958242778555	-0.123366058393418	-0.128015140306531
-0.513200713701101	-0.494288182492599	-0.463066080330132	0.248738336882847	0.222509478261147	0.171382431250614	-0.287746623847822	-0.328072621380567	-0.398843965596179	-0.00812986327651255	-0.0273155532108618	-0.0292019093116052	0.163374947098307	-0.028120328258814	0.105723096530503	0.0817175876343479
0.51491261519899	0.451791352047035	0.366991016765645	-0.0778712157070734	-0.0460954149519256	0.0115920150305794	0.454087650211114	0.526057678507092	0.618352330610155	0.352971798697582	0.344618844875416	0.360042088672109	-0.453166486987061	-0.0237707937060184	-0.253880064048325	-0.243860573382503
0.448602950612171	0.384868212505539	0.302087778450658	-0.0579652149001027	-0.0220217730203188	0.0293744312135818	0.435760512927589	0.507131418419285	0.595774203218544	0.401988330241274	0.392858046161703	0.408738525790329	-0.488158891006741	-0.0117150163073688	-0.302251243452368	-0.287339661647386
-0.547532449990338	-0.503549941776979	-0.441019787314524	0.210563563374025	0.17272031371366	0.122639398684627	-0.351112017286208	-0.41588691089752	-0.498828004058587	-0.0578439650559196	-0.0667619557742531	-0.0702517207538805	0.394839421931676	0.129961553432005	0.260296249392073	0.233371769358853
0.57768016286954	0.539626686057041	0.491187788247322	-0.170051867428834	-0.144286418900512	-0.0922598091270205	0.290230725353338	0.33536414486843	0.418781988225422	0.03857939351178	0.0606274690722593	0.0616769976277098	-0.469531740359617	-0.189476829660698	-0.337741266392666	-0.304434353673408
-0.719257123215272	-0.679364594005807	-0.603539476318788	0.0606967563823985	0.0381228939552023	-0.0340288340708465	-0.522897841870012	-0.562106326576402	-0.650637113392348	-0.017994566245785	-0.0170014306454433	-0.0251173605113637	0.174943510844535	0.0382647140324277	0.0190202511194567	0.021832377107426
0.75572016123453	0.724144291315	0.669295900619896	-0.167267578729981	-0.134563527429365	-0.0670093027642	0.347807029047135	0.383561513091342	0.477357658941595	-0.00795576954173528	0.00741069390517613	0.0104661014725306	-0.253211456529905	-0.0133556280740898	-0.0467012373674046	-0.0317891665986029
0.675863474563473	0.671597677479998	0.657922606409146	-0.219330517466142	-0.158904228513119	-0.109350962650882	0.145959679446742	0.176590157328717	0.25314026336462	-0.178123762011261	-0.155502348929719	-0.157431788718771	-0.333547862591875	-0.0487595031885931	-0.0622085898645183	-0.0432868094417676
-0.552917127920233	-0.498112652321224	-0.419357979568814	0.0552434814623059	0.0633296063399375	0.00128126386902462	-0.477500559476945	-0.517498100702652	-0.598866212464643	-0.144981771617033	-0.152323387247365	-0.158518708737828	0.318097303280321	0.171039945361379	0.279520294420961	0.258830964528397
0.680576300529822	0.640555600626177	0.579745016747922	-0.134400085980403	-0.11359792087319	-0.0487243828869442	0.396280589941215	0.43752519155791	0.527658084846243	0.0230209690535185	0.0380272586369655	0.0414964506479823	-0.367359349198612	-0.140406037584439	-0.221812719933518	-0.199132077571426
1	0.991489566383089	0.971364086331252	-0.0340062428019166	-0.0579929845660317	0.0295792597032972	0.378930273035002	0.3564245831692	0.429945825469564	-0.107867682832017	-0.106169909927742	-0.0959265553817255	-0.143764897012411	0.191837971146918	0.132457548810113	0.160740101730704
0.991489566383089	1	0.985805524653313	-0.0186010516203434	-0.0482609015014779	0.0362061823733118	0.355533235618293	0.329809562866212	0.392929469976217	-0.138698726685154	-0.13636287771887	-0.126186369585749	-0.11989272063487	0.199762925550082	0.157323814126763	0.187606191914925
0.971364086331252	0.985805524653313	1	-0.0386993649405419	-0.0771142670975222	0.00730475071667745	0.279256605966796	0.239671229791127	0.294231949238681	-0.192201118069054	-0.186710988140251	-0.178499994841828	-0.114933731908111	0.194374545752918	0.173444729436443	0.197861895362077
-0.0340062428019166	-0.0186010516203434	-0.0386993649405419	1	0.874243216310205	0.87773070187142	0.532441429610887	0.48161040235873	0.33757813108043	0.365015411463866	0.365085613331198	0.37560370624603	0.348262285424103	0.485373726176603	0.417292811439778	0.423293212289065
-0.0579929845660317	-0.0482609015014779	-0.0771142670975222	0.874243216310205	1	0.976474254308027	0.551059775848423	0.525105133102692	0.403053443483035	0.221472750613324	0.233371343588171	0.23358855027593	0.302858642019352	0.557807611160507	0.336225415333232	0.353375226345274
0.0295792597032972	0.0362061823733118	0.00730475071667745	0.87773070187142	0.976474254308027	1	0.603669403393568	0.565254622031554	0.446894996549572	0.204882574337537	0.215496512086033	0.221786402768033	0.302429894952057	0.588117828113248	0.348094976902045	0.382539327644667
0.378930273035002	0.355533235618293	0.279256605966796	0.532441429610887	0.551059775848423	0.603669403393568	1	0.97925826340914	0.940235988570284	0.325481902559511	0.319936701613471	0.334193461859056	0.277857857946856	0.421964983000786	0.231045340392759	0.26054907762448
0.3564245831692	0.329809562866212	0.239671229791127	0.48161040235873	0.525105133102692	0.565254622031554	0.97925826340914	1	0.974362029461248	0.355481744535925	0.349164674381301	0.363139759038848	0.213843100973241	0.36491152429515	0.178303942862719	0.193941360533626
0.429945825469564	0.392929469976217	0.294231949238681	0.33757813108043	0.403053443483035	0.446894996549572	0.940235988570284	0.974362029461248	1	0.333411592933792	0.325603450115007	0.340079977306687	0.127612019460458	0.294763520653157	0.103383313659759	0.116395817409136
-0.107867682832017	-0.138698726685154	-0.192201118069054	0.365015411463866	0.221472750613324	0.204882574337537	0.325481902559511	0.355481744535925	0.333411592933792	1	0.997426083231281	0.996637812445254	0.0559599308879003	0.0472844070507482	-0.00810385647689264	-0.0321316851865526
-0.106169909927742	-0.13636287771887	-0.186710988140251	0.365085613331198	0.233371343588171	0.215496512086033	0.319936701613471	0.349164674381301	0.325603450115007	0.997426083231281	1	0.997226207301223	0.0561567657178863	0.0478134647707505	-0.0149098324553526	-0.0353028579354924
-0.0959265553817255	-0.126186369585749	-0.178499994841828	0.37560370624603	0.23358855027593	0.221786402768033	0.334193461859056	0.363139759038848	0.340079977306687	0.996637812445254	0.997226207301223	1	0.0472475459391368	0.0550818259227493	-0.0111523528031292	-0.031073862864249
-0.143764897012411	-0.11989272063487	-0.114933731908111	0.348262285424103	0.302858642019352	0.302429894952057	0.277857857946856	0.213843100973241	0.127612019460458	0.0559599308879003	0.0561567657178863	0.0472475459391368	1	0.454255088947894	0.650585833039377	0.669930575363032
0.191837971146918	0.199762925550082	0.194374545752918	0.485373726176603	0.557807611160507	0.588117828113248	0.421964983000786	0.36491152429515	0.294763520653157	0.0472844070507482	0.0478134647707505	0.0550818259227493	0.454255088947894	1	0.614617974356204	0.673935118447933
0.132457548810113	0.157323814126763	0.173444729436443	0.417292811439778	0.336225415333232	0.348094976902045	0.231045340392759	0.178303942862719	0.103383313659759	-0.00810385647689264	-0.0149098324553526	-0.0111523528031292	0.650585833039377	0.614617974356204	1	0.963593312316837
0.160740101730704	0.187606191914925	0.197861895362077	0.423293212289065	0.353375226345274	0.382539327644667	0.26054907762448	0.193941360533626	0.116395817409136	-0.0321316851865526	-0.0353028579354924	-0.031073862864249	0.669930575363032	0.673935118447933	0.963593312316837	1
-0.23275530982033	-0.219230080360153	-0.194654808150825	0.289157311062092	0.249194516148197	0.251229459475031	0.0494389476731126	-0.0367447802265318	-0.11097217231455	0.0953467923328756	0.0967915828161188	0.0957369006080973	0.625785047138532	0.513945689868949	0.443845734478057	0.492020833383151
-0.169721156943375	-0.163957373703801	-0.149790202451084	0.269985424313147	0.250066404220886	0.270976227090781	0.150119135889959	0.0506562538453257	-0.0142980579272863	0.0722729760269093	0.0726226340358131	0.0733026298345233	0.58850041952262	0.536441276601955	0.367929853074081	0.456008046883837
-0.611881796353813	-0.575822502175061	-0.539346843253684	0.277621748435412	0.284440030437725	0.18414621058759	-0.195371783909303	-0.219999770901695	-0.32403773327994	0.0379723644993262	0.0286541890119417	0.00581280610850226	0.569232690687453	0.190687032168833	0.392908120684462	0.313853181998349
-0.434088493735299	-0.413904954403846	-0.410948094372436	0.29191496481494	0.34330930379644	0.271401918250083	0.049340063900817	0.0388962841453821	-0.0641363027212567	0.0348232921561765	0.0239950758259233	0.00499054456760032	0.681148930351999	0.369170034581796	0.449583501401673	0.405649948112782
-0.364104231234498	-0.355003863111465	-0.365301668318661	0.314278633350112	0.377926928621517	0.33395784771691	0.195254272656716	0.168498152084893	0.0930318175575476	0.0607345793039973	0.0459070267853952	0.0311378017168993	0.629835557107463	0.315737388322726	0.397551137858181	0.369154394508144
-0.0465827602695972	-0.0240144603501955	0.0414597569209873	0.0900286297138507	-0.0561968362276506	-0.0947598252661347	-0.300300466134295	-0.355130240036154	-0.410190650821	-0.121732132774592	-0.109625162034305	-0.119052086356448	0.15348962308314	0.0475506468140576	0.270801893678604	0.258450902215525
0.105302591510784	0.137538158933557	0.212544267771429	0.171928569038483	0.0171974581281049	-0.00195540430484701	-0.188918436381282	-0.251630743999546	-0.312960568860352	-0.133836516237668	-0.120146941104897	-0.123643314847116	0.170576349269503	0.121201478342703	0.311870161863763	0.291166034399999
0.0851119944299155	0.114224778214	0.178807886272515	0.290062102637979	0.135173155932018	0.119598979160942	-0.127306909401592	-0.206752815662516	-0.284630708539084	-0.114741436364896	-0.101933073526968	-0.105451216486578	0.229706042422424	0.208906467823753	0.423838942425017	0.41881281087047
0.557492460599371	0.519798466998621	0.450733764173553	-0.0219542218807627	0.0633901585951348	0.121800363305139	0.591015735134816	0.602607127150994	0.657769463148607	-0.169973007046217	-0.173321790244977	-0.171457436101027	0.0251729438730149	0.0941868397838604	0.0389948738311235	0.043380285625553
0.605927021083064	0.573945540987581	0.507683958320593	0.0452183927369138	0.0970242016717819	0.164336777571411	0.667149341859646	0.662453090441303	0.708671615276761	-0.125951593307178	-0.126980178399236	-0.125809640664653	0.0939937270284308	0.119921049837625	0.0562796667497074	0.0794940531527404
0.610594675584243	0.576101749836181	0.506072885620789	0.081253463603843	0.128709344529886	0.199660668748672	0.684668680715942	0.679279631907876	0.7233298618355	-0.0971340296746015	-0.0992082955306855	-0.0959861085816885	0.0833998774240167	0.140390014516844	0.0704036780731525	0.095963311040447
-0.529983460367735	-0.500632686566114	-0.422843660437636	-0.312565997291504	-0.356537195999517	-0.41832492829564	-0.910085833866768	-0.901898364083447	-0.905898763164518	-0.0960963772298136	-0.0927556628717615	-0.101516079379198	-0.19317890994435	-0.292508629237	-0.150943878974095	-0.177831443643364
-0.513257846204631	-0.478944093245658	-0.391926966684625	-0.297144129569107	-0.349785896067913	-0.407977754040281	-0.910321184154	-0.919063349637556	-0.929782454217188	-0.141396060132313	-0.13736523327945	-0.146058167853995	-0.173458963686015	-0.268990277668271	-0.127122392404024	-0.150976422445291
-0.55547640781269	-0.51882518566214	-0.428007935613943	-0.23714910424374	-0.298253057543087	-0.358991058285143	-0.884673998977085	-0.900538270289507	-0.933648997972984	-0.146747015237732	-0.141400729290903	-0.150510280470242	-0.121045415498369	-0.245726944541312	-0.0968086809603297	-0.117968030200674

exkx_d1	exkx_d2	orcdrc_d1	orcdrc_d2	orcdrc_d3	phihox_d1	phihox_d2	phihox_d3	sltppt_d1	sltppt_d2	sltppt_d3	sndppt_d1	sndppt_d2	sndppt_d3
-0.139341089233197	-0.16282880618948	-0.158563655681715	-0.0786693683552278	-0.0927606969642114	0.28887966669154	0.373557805044893	0.341499184581521	0.232008636646802	0.212294467697783	0.192059196662323	-0.0957497662124756	-0.0517204112538598	-0.0660230528948089
-0.341475842503002	-0.323498508048356	-0.494188598949919	-0.327809538611576	-0.308036307798098	0.0855104531340121	0.195221730283765	0.156274824335811	0.563591686679406	0.548653117418333	0.532018923963168	-0.404691030106469	-0.382518957544408	-0.415703221753498
-0.0442309200125858	-0.0633469817146399	-0.0254384636412572	0.0146497488602977	0.0343756228210862	0.301546969096923	0.353042527192001	0.343849576406944	0.128332870556178	0.116238801724913	0.0983012081326863	-0.0434699217157918	0.00296171282780853	-0.000458167930197022
0.194654169402331	0.133887941716703	0.343024686527785	0.188145775451055	0.107467843456584	-0.0384780420212017	-0.122520977527948	-0.0849514347512398	-0.610481796326491	-0.657879226773966	-0.624652236972495	0.52810469018415	0.505361812881634	0.516760767956027
-0.424908974669841	-0.331237339433273	-0.621920787546661	-0.391559032675994	-0.29053681065566	-0.437031281047383	-0.3571712281156	-0.370073037201218	0.620752721987418	0.612354437788979	0.641601366003185	-0.624963151424098	-0.66513385349088	-0.720265269971371
0.430057777106955	0.385297633349865	0.600421174910789	0.360848494300371	0.407771201223569	0.0408101739592995	-0.0546194593365677	0.0313155634490795	-0.551866243223664	-0.563087819010142	-0.54557614389598	0.474119657688967	0.484655089414679	0.512402046868939
-0.489378147652451	-0.400470977897709	-0.698962601092968	-0.491375019986821	-0.434121028017781	-0.390295794187404	-0.322148610404853	-0.35163828708426	0.567888460007792	0.546168202641261	0.57013567379532	-0.550298561388372	-0.591799419504989	-0.644318942829863
-0.466159980139231	-0.378226149788675	-0.676727028525406	-0.488805194722558	-0.425952214881605	-0.409372586134546	-0.344720673257443	-0.373487390976167	0.489986616518336	0.469055060977026	0.496312238985598	-0.497851581837056	-0.541597121333326	-0.59377153084622
0.615125375953696	0.519559570280437	0.748380436182492	0.521859738054821	0.498121877847377	0.128246503529561	0.0620011694864595	0.124277748789019	-0.660650856229402	-0.651996258245531	-0.643172501405011	0.563621078796001	0.582711709932318	0.612898410628796
-0.569374418326746	-0.496624966236669	-0.784390518274887	-0.633623258617535	-0.609938677960685	-0.150205050354638	-0.0657402232925179	-0.141546963637894	0.620961678415923	0.617618725419516	0.607565775198927	-0.506900632293837	-0.515784438028875	-0.550469974884735
0.419135096461354	0.348018212351145	0.607532926005118	0.385588349404957	0.29564293985478	0.326864070844836	0.241633408546787	0.249080698641329	-0.81183142705767	-0.811370732426622	-0.816685514479193	0.723045138870425	0.746721440500032	0.782168770046673
-0.403993389184503	-0.357083402987599	-0.674632247949279	-0.478715449794668	-0.462048569748855	-0.199215210502576	-0.088347009920418	-0.134789790950369	0.68612537665873	0.682664651767945	0.677233223068292	-0.561276465983118	-0.574963475938009	-0.618489720122995
-0.519885371454368	-0.482599515768546	-0.672795464237311	-0.522391573488911	-0.516693402019397	0.0848205858238864	0.188380953316376	0.116649135005346	0.547868128148966	0.529340233023995	0.509017584603615	-0.389912830395938	-0.375879758231839	-0.407997609266023
0.427080415307583	0.344171829903222	0.666383050779705	0.495835838585903	0.431876893532842	0.379837973018285	0.311322759270331	0.342518058753456	-0.717073792376984	-0.728333365319373	-0.732957490574233	0.651360593344032	0.679165903126689	0.712087509921694
-0.5247410211296	-0.450710647736703	-0.758229008684452	-0.573704656079527	-0.526684085071892	-0.227340062659016	-0.134340481641965	-0.187435557750999	0.73887399113531	0.738608396339743	0.732938155684684	-0.624031700328379	-0.637251338564995	-0.674220298351919
-0.23275530982033	-0.169721156943375	-0.611881796353813	-0.434088493735299	-0.364104231234498	-0.0465827602695972	0.105302591510784	0.0851119944299155	0.557492460599371	0.605927021083064	0.610594675584243	-0.529983460367735	-0.513257846204631	-0.55547640781269
-0.219230080360153	-0.163957373703801	-0.575822502175061	-0.413904954403846	-0.355003863111465	-0.0240144603501955	0.137538158933557	0.114224778214	0.519798466998621	0.573945540987581	0.576101749836181	-0.500632686566114	-0.478944093245658	-0.51882518566214
-0.194654808150825	-0.149790202451084	-0.539346843253684	-0.410948094372436	-0.365301668318661	0.0414597569209873	0.212544267771429	0.178807886272515	0.450733764173553	0.507683958320593	0.506072885620789	-0.422843660437636	-0.391926966684625	-0.428007935613943
0.289157311062092	0.269985424313147	0.277621748435412	0.29191496481494	0.314278633350112	0.0900286297138507	0.171928569038483	0.290062102637979	-0.0219542218807627	0.0452183927369138	0.081253463603843	-0.312565997291504	-0.297144129569107	-0.23714910424374
0.249194516148197	0.250066404220886	0.284440030437725	0.34330930379644	0.377926928621517	-0.0561968362276506	0.0171974581281049	0.135173155932018	0.0633901585951348	0.0970242016717819	0.128709344529886	-0.356537195999517	-0.349785896067913	-0.298253057543087
0.251229459475031	0.270976227090781	0.18414621058759	0.271401918250083	0.33395784771691	-0.0947598252661347	-0.00195540430484701	0.119598979160942	0.121800363305139	0.164336777571411	0.199660668748672	-0.41832492829564	-0.407977754040281	-0.358991058285143
0.0494389476731126	0.150119135889959	-0.195371783909303	0.049340063900817	0.195254272656716	-0.300300466134295	-0.188918436381282	-0.127306909401592	0.591015735134816	0.667149341859646	0.684668680715942	-0.910085833866768	-0.910321184154	-0.884673998977085
-0.0367447802265318	0.0506562538453257	-0.219999770901695	0.0388962841453821	0.168498152084893	-0.355130240036154	-0.251630743999546	-0.206752815662516	0.602607127150994	0.662453090441303	0.679279631907876	-0.901898364083447	-0.919063349637556	-0.900538270289507
-0.11097217231455	-0.0142980579272863	-0.32403773327994	-0.0641363027212567	0.0930318175575476	-0.410190650821	-0.312960568860352	-0.284630708539084	0.657769463148607	0.708671615276761	0.7233298618355	-0.905898763164518	-0.929782454217188	-0.933648997972984
0.0953467923328756	0.0722729760269093	0.0379723644993262	0.0348232921561765	0.0607345793039973	-0.121732132774592	-0.133836516237668	-0.114741436364896	-0.169973007046217	-0.125951593307178	-0.0971340296746015	-0.0960963772298136	-0.141396060132313	-0.146747015237732
0.0967915828161188	0.0726226340358131	0.0286541890119417	0.0239950758259233	0.0459070267853952	-0.109625162034305	-0.120146941104897	-0.101933073526968	-0.173321790244977	-0.126980178399236	-0.0992082955306855	-0.0927556628717615	-0.13736523327945	-0.141400729290903
0.0957369006080973	0.0733026298345233	0.00581280610850226	0.00499054456760032	0.0311378017168993	-0.119052086356448	-0.123643314847116	-0.105451216486578	-0.171457436101027	-0.125809640664653	-0.0959861085816885	-0.101516079379198	-0.146058167853995	-0.150510280470242
0.625785047138532	0.58850041952262	0.569232690687453	0.681148930351999	0.629835557107463	0.15348962308314	0.170576349269503	0.229706042422424	0.0251729438730149	0.0939937270284308	0.0833998774240167	-0.19317890994435	-0.173458963686015	-0.121045415498369
0.513945689868949	0.536441276601955	0.190687032168833	0.369170034581796	0.315737388322726	0.0475506468140576	0.121201478342703	0.208906467823753	0.0941868397838604	0.119921049837625	0.140390014516844	-0.292508629237	-0.268990277668271	-0.245726944541312
0.443845734478057	0.367929853074081	0.392908120684462	0.449583501401673	0.397551137858181	0.270801893678604	0.311870161863763	0.423838942425017	0.0389948738311235	0.0562796667497074	0.0704036780731525	-0.150943878974095	-0.127122392404024	-0.0968086809603297
0.492020833383151	0.456008046883837	0.313853181998349	0.405649948112782	0.369154394508144	0.258450902215525	0.291166034399999	0.41881281087047	0.043380285625553	0.0794940531527404	0.095963311040447	-0.177831443643364	-0.150976422445291	-0.117968030200674
1	0.96155658486837	0.552857838557622	0.50207318941554	0.474816384074965	0.0741505798566119	0.039748991957217	0.110327661932169	-0.220583231992901	-0.214122783804993	-0.2055967765125	0.109200952685727	0.122274188359327	0.167583300733043
0.96155658486837	1	0.443914924035882	0.434961052802699	0.454743440924149	0.053719034660427	0.0254925518556492	0.0941384887480658	-0.127169509858296	-0.105964416861848	-0.0982757650134113	-0.00423774365117624	0.0141107965567459	0.0583693356763185
0.552857838557622	0.443914924035882	1	0.873978202547966	0.742638143988991	0.209047654675164	0.145021884686825	0.179802068682409	-0.444505441943304	-0.459422689873682	-0.46767414297227	0.359666349456591	0.36505757256071	0.417541528940213
0.50207318941554	0.434961052802699	0.873978202547966	1	0.859164932495533	0.15647268254866	0.130518896949481	0.14494225626173	-0.187077545258022	-0.182725903943561	-0.185419480096395	0.0729364316032682	0.0715477412365793	0.123892308217605
0.474816384074965	0.454743440924149	0.742638143988991	0.859164932495533	1	0.0842180493364848	0.0484880797465703	0.0490584973414731	-0.107952922825719	-0.0674589700892478	-0.0626995346172271	-0.0673798854747206	-0.0658922324048859	-0.0239751285328887
0.0741505798566119	0.053719034660427	0.209047654675164	0.15647268254866	0.0842180493364848	1	0.933160474825554	0.901475603433948	-0.277495859015469	-0.238744097116926	-0.254869720111521	0.282397814565222	0.329289195361322	0.361949706071716
0.039748991957217	0.0254925518556492	0.145021884686825	0.130518896949481	0.0484880797465703	0.933160474825554	1	0.943197459719743	-0.190475287645168	-0.136649573981544	-0.157537006225352	0.163276355134236	0.221574164678414	0.251957979646514
0.110327661932169	0.0941384887480658	0.179802068682409	0.14494225626173	0.0490584973414731	0.901475603433948	0.943197459719743	1	-0.21448145512539	-0.160450099677233	-0.168398050050221	0.147339651791963	0.205785229396932	0.244268355684132
-0.220583231992901	-0.127169509858296	-0.444505441943304	-0.187077545258022	-0.107952922825719	-0.277495859015469	-0.190475287645168	-0.21448145512539	1	0.954713597938523	0.950374208644687	-0.850508548161967	-0.847629739174681	-0.856724358664477
-0.214122783804993	-0.105964416861848	-0.459422689873682	-0.182725903943561	-0.0674589700892478	-0.238744097116926	-0.136649573981544	-0.160450099677233	0.954713597938523	1	0.99478831492421	-0.910763088908703	-0.901726515684084	-0.910621126915071
-0.2055967765125	-0.0982757650134113	-0.46767414297227	-0.185419480096395	-0.0626995346172271	-0.254869720111521	-0.157537006225352	-0.168398050050221	0.950374208644687	0.99478831492421	1	-0.916455714723001	-0.910216988151945	-0.920504772460726
0.109200952685727	-0.00423774365117624	0.359666349456591	0.0729364316032682	-0.0673798854747206	0.282397814565222	0.163276355134236	0.147339651791963	-0.850508548161967	-0.910763088908703	-0.916455714723001	1	0.993488100842471	0.982923608413937
0.122274188359327	0.0141107965567459	0.36505757256071	0.0715477412365793	-0.0658922324048859	0.329289195361322	0.221574164678414	0.205785229396932	-0.847629739174681	-0.901726515684084	-0.910216988151945	0.993488100842471	1	0.992499432778867
0.167583300733043	0.0583693356763185	0.417541528940213	0.123892308217605	-0.0239751285328887	0.361949706071716	0.251957979646514	0.244268355684132	-0.856724358664477	-0.910621126915071	-0.920504772460726	0.982923608413937	0.992499432778867	1

Supplementary Figures S1, S2, S3 and S4

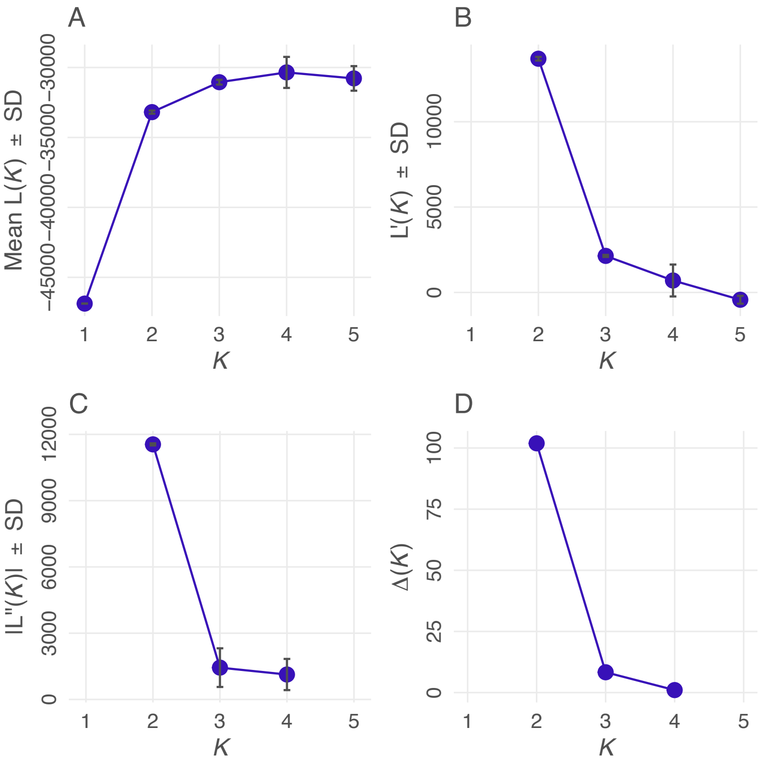

Fig. S1. Evanno output plots.

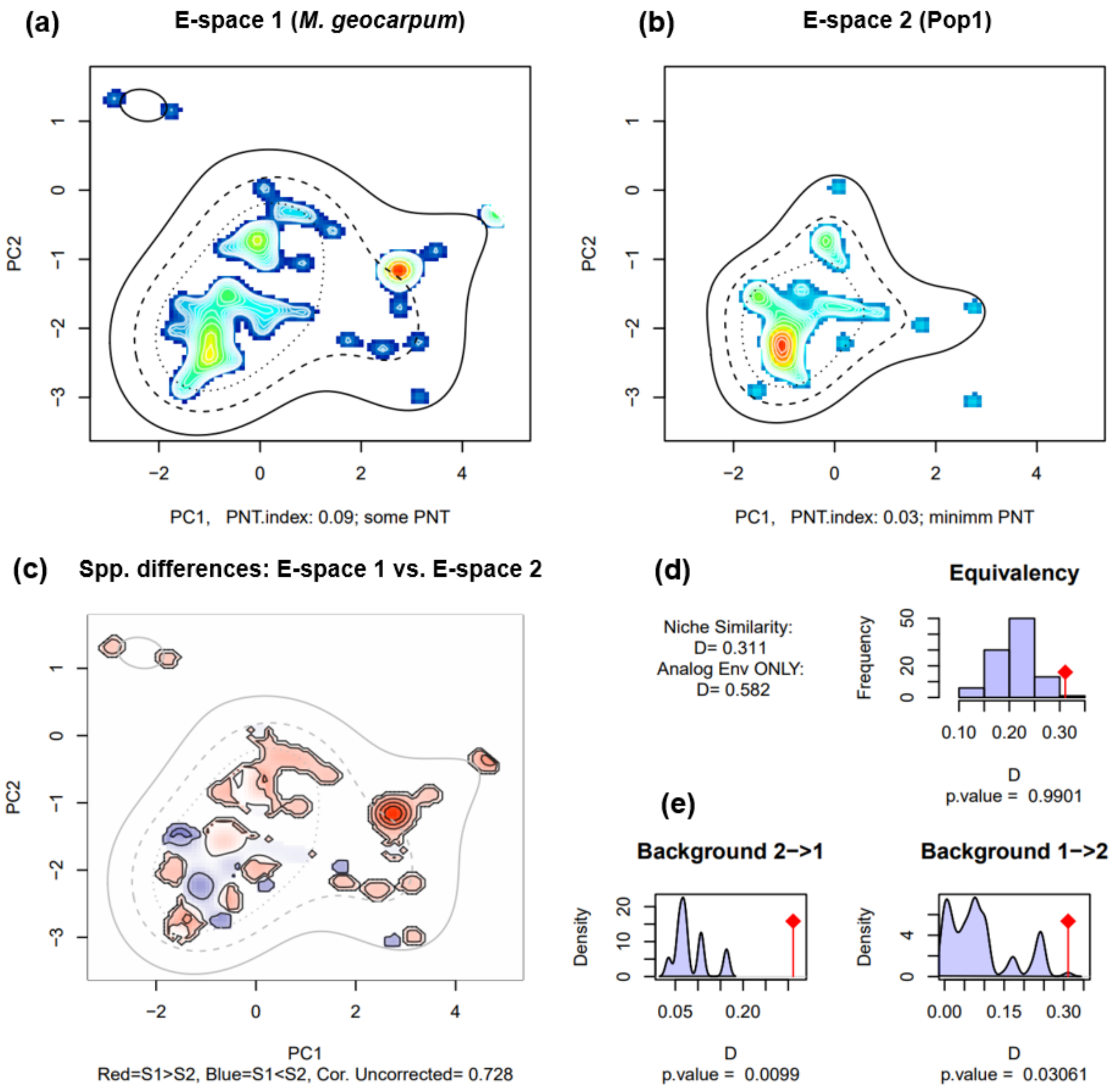

Fig. S2. Niche equivalency and niche background tests between *M. geocarpum* and genetic Pop1. Graphs **(a)** and **(b)** represent the kernel density isopleths, red color indicates high density and blue colour indicates low density. **(a)** and **(b)** represent also the Potential Niche Truncation Index (PNTI) describing the amount of observed E-space of the groups that is truncated by the available E-space. **(c)** represents the difference in the E-space of two compared groups and Niche E-space Correlation Index. **(d)** represents the Equivalency statistic measured as Niche similarity index and **(e)** represents niche Background statistic.

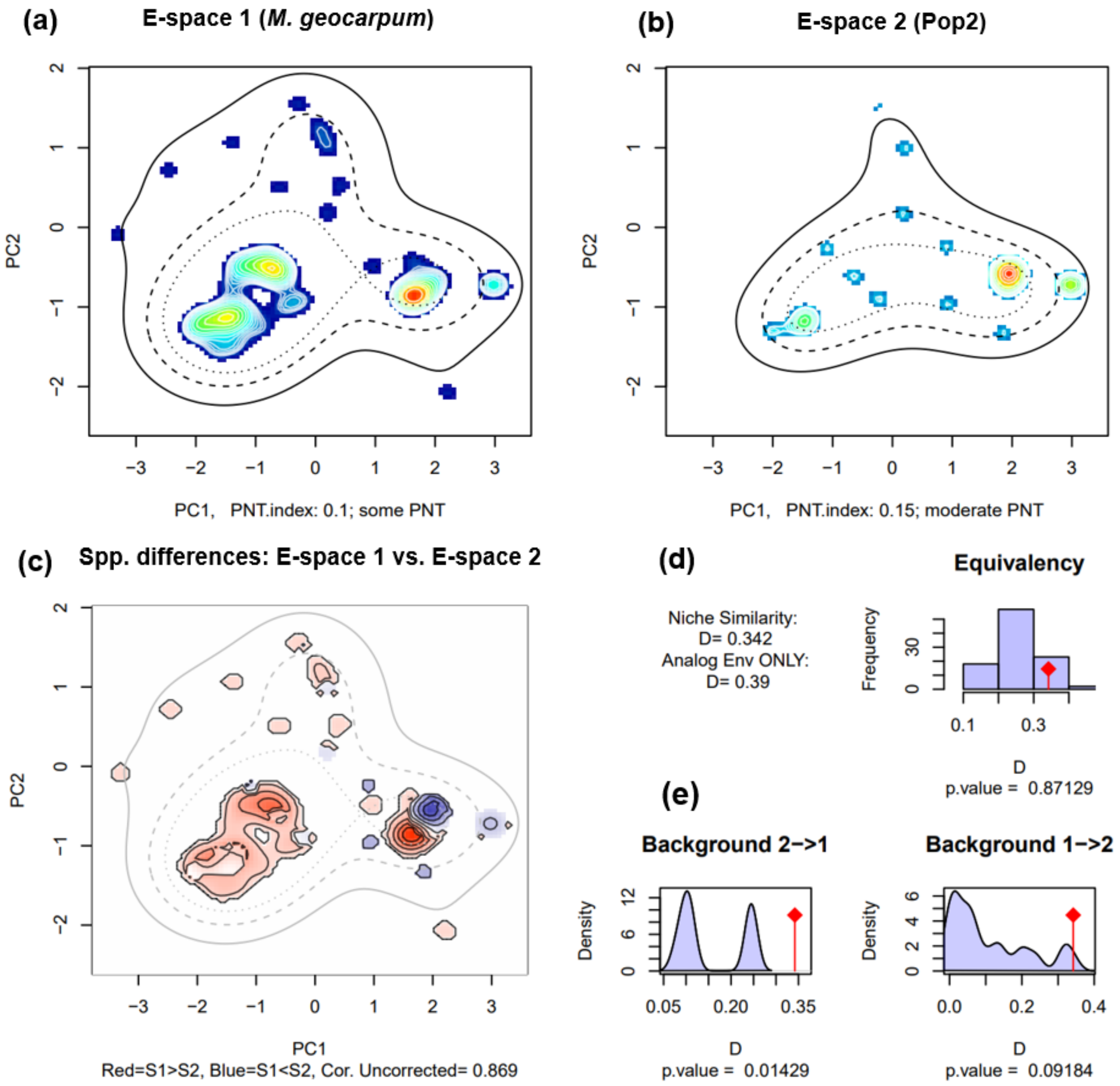

Fig. S3. Niche equivalency and niche background tests between *M. geocarpum* and genetic Pop2. Graphs **(a)** and **(b)** represent the kernel density isopleths, red color indicates high density and blue colour indicates low density. **(a)** and **(b)** represent also the Potential Niche Truncation Index (PNTI) describing the amount of observed E-space of the groups that is truncated by the available E-space. **(c)** represents the difference in the E-space of two groups and Niche E-space Correlation Index. **(d)** represents the Equivalency statistic measured as Niche similarity index and **(e)** represents niche Background statistic.

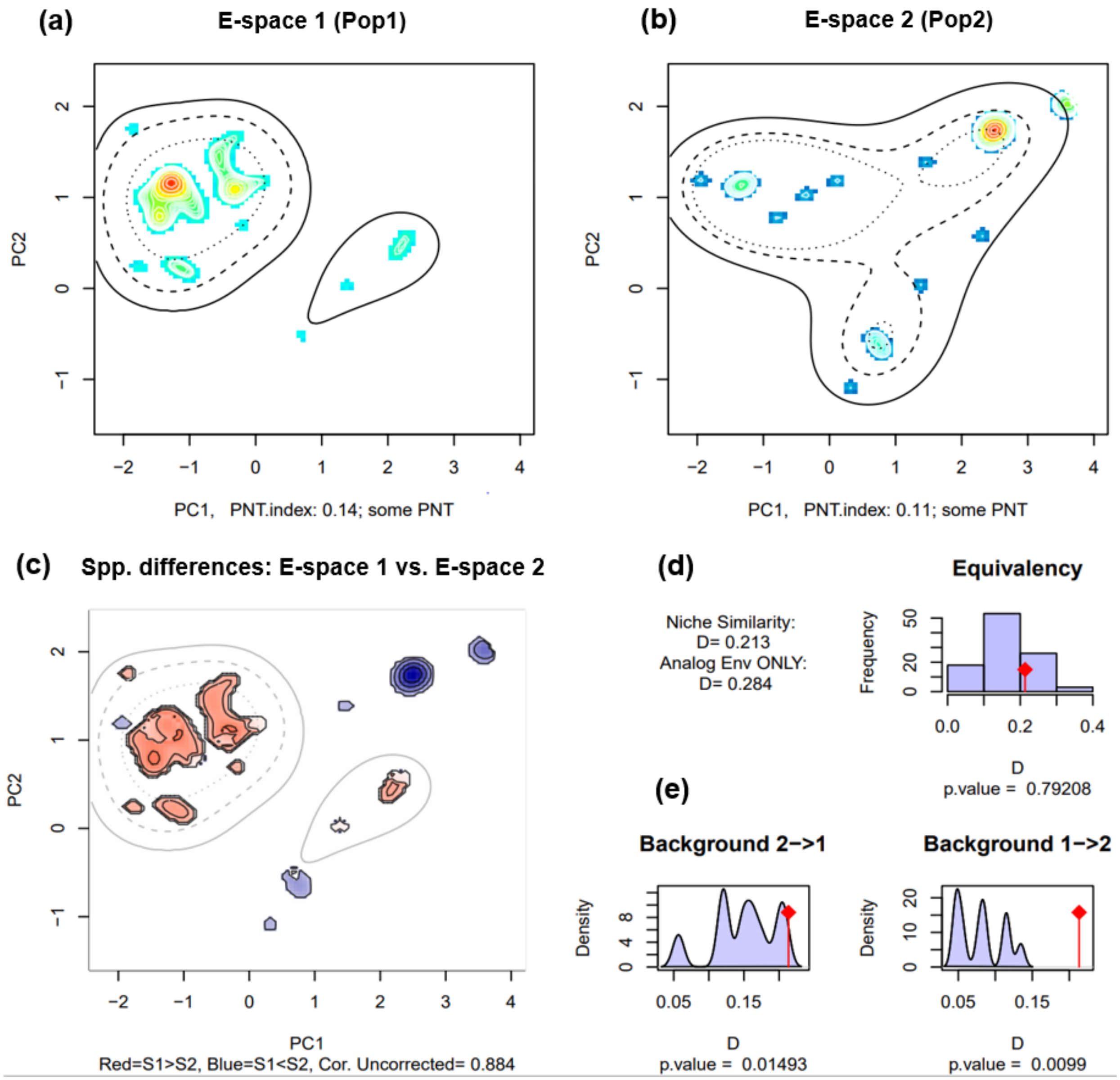

Fig. S4. Niche equivalency and niche background tests between the two genetic populations. Graphs **(a)** and **(b)** represent the kernel density isopleths, red color indicates high density and blue colour indicates low density. **(a)** and **(b)** represent also the Potential Niche Truncation Index (PNTI) describing the amount of observed E-space of the populations that is truncated by the available E-space. **(c)** represents the difference in the E-space of two populations and Niche E-space Correlation Index. **(d)** represents the Equivalency statistic measured as Niche similarity index and **(e)** represents niche Background statistic.

Supplementary Figures S5, S6 and S7

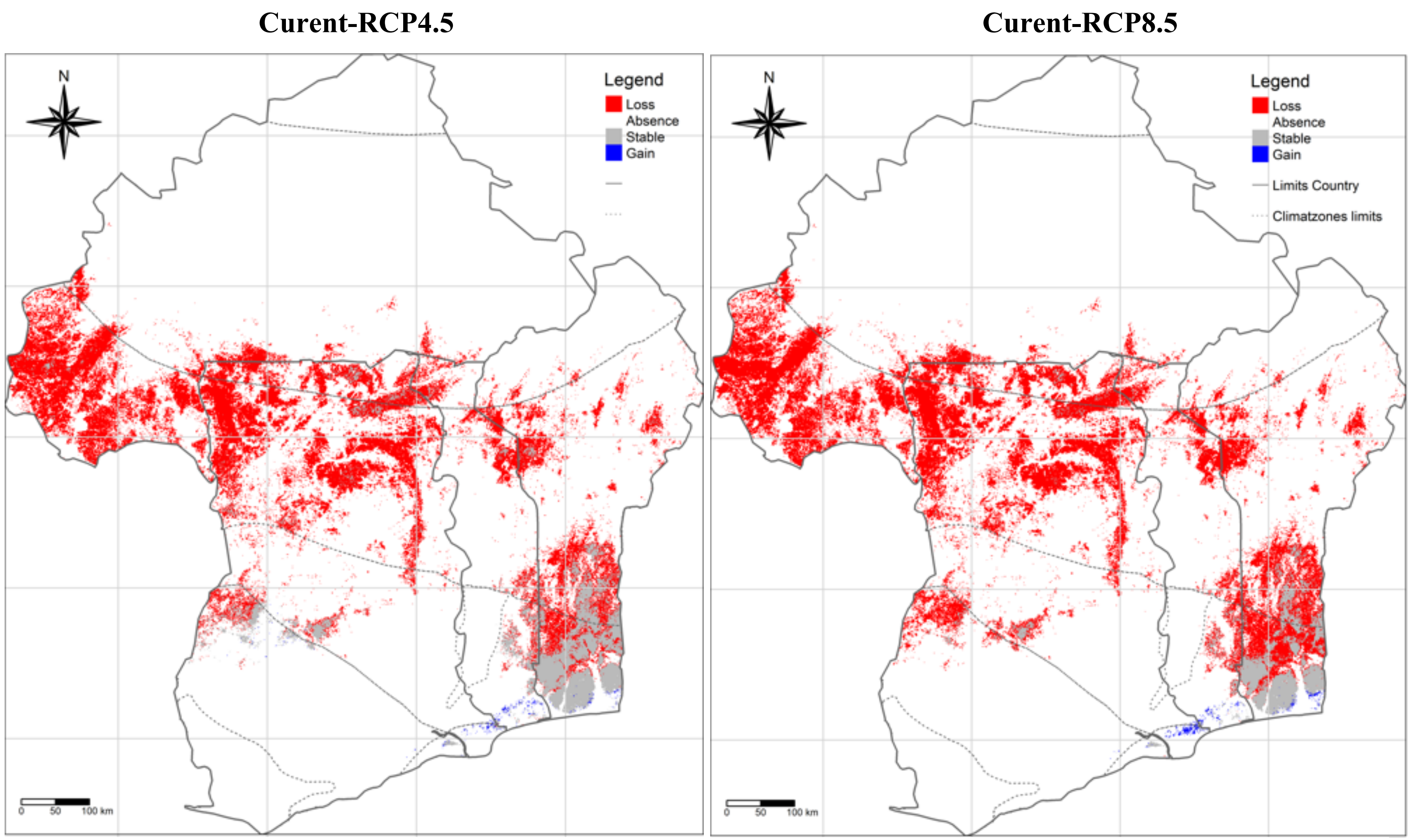

Fig. S5. Maps showing the projected future cultivable areas of *M geocarpum*, classified into gain, stable and loss categories deriving from comparing cultivable areas in the current time and future projections (RCP4.5 and RCP8.5). Software used: package "tmap" version 3.3-1 in R.

Curent-RCP4.5

Curent-RCP8.5

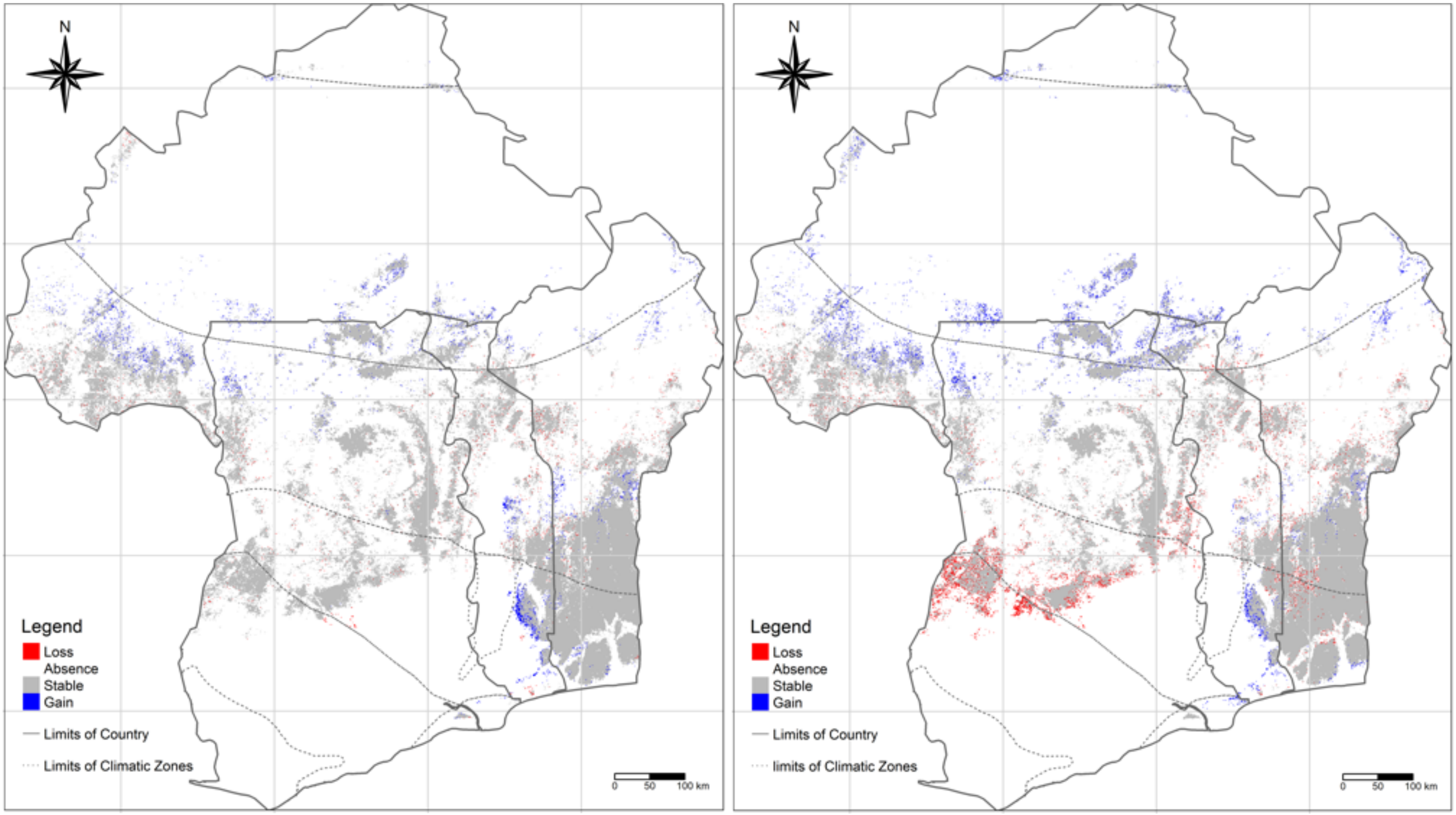

Fig. S6. Maps showing the projected future cultivable areas of Genetic Population 1, classified into gain, stable and loss categories deriving from comparing cultivable areas in the current time and future projections (RCP4.5 and RCP8.5). Software used: package "tmap" version 3.3-1 in R.

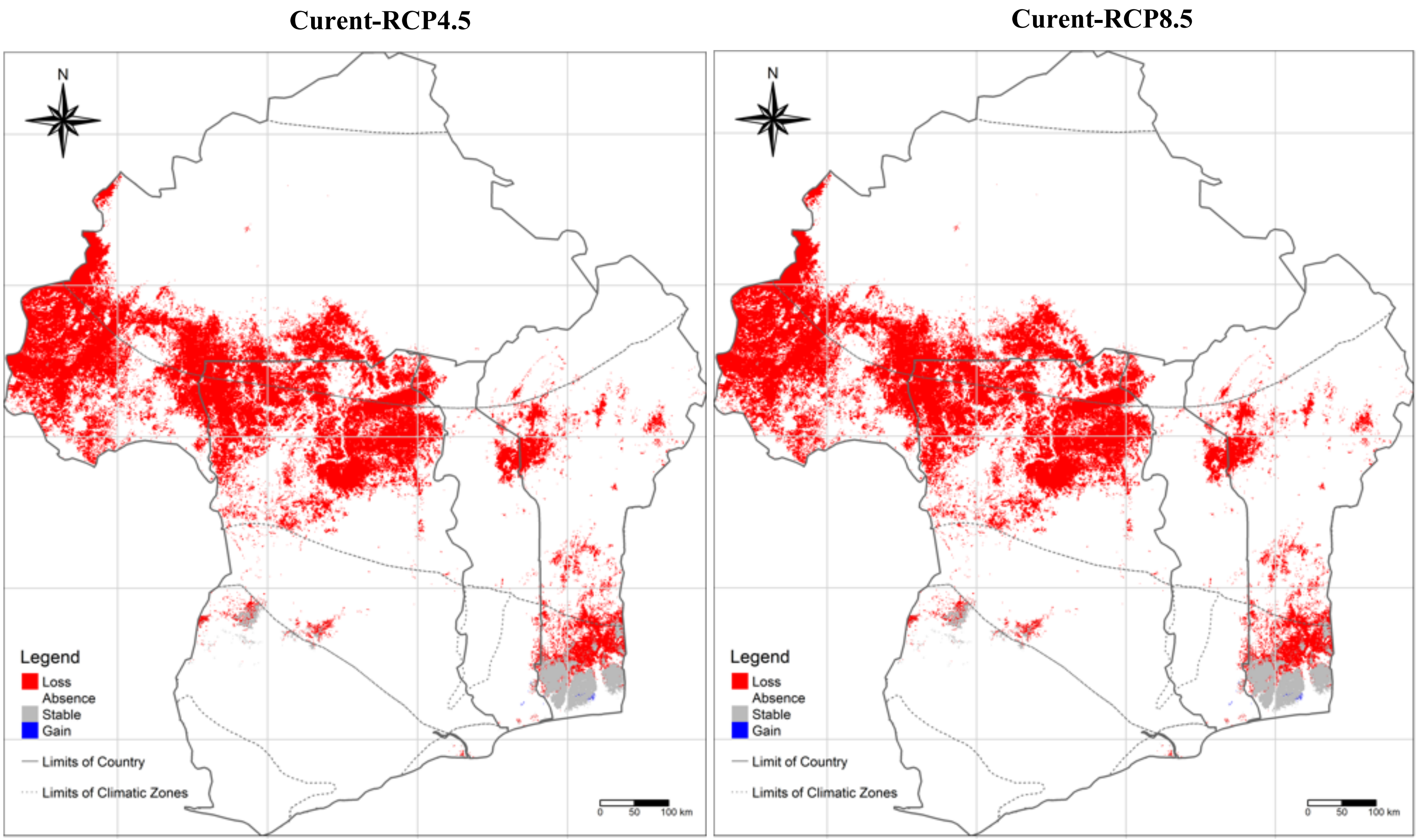

Fig. S7. Maps showing the projected future cultivable areas of Genetic Population 2, classified into gain, stable and loss categories deriving from comparing cultivable areas in the current time and future projections (RCP4.5 and RCP8.5). Software used: package "tmap" version 3.3-1 in R.
